# Interaction of a 1,3-Dicarbonyl Toxin with Ru(II)-Biimidazole Complexes for Luminescence Sensing: A Spectroscopic and Photochemical Experimental Study Rationalized by Time-Dependent Density Functional Theory Calculations

**DOI:** 10.1021/acs.inorgchem.1c02887

**Published:** 2021-12-19

**Authors:** José Quílez-Alburquerque, Cristina García-Iriepa, Marco Marazzi, Ana B. Descalzo, Guillermo Orellana

**Affiliations:** † Department of Organic Chemistry, Faculty of Chemistry, Universidad Complutense de Madrid, Madrid 28040, Spain; ‡ Departamento de Química Analítica, Química Física e Ingeniería Química, Universidad de Alcalá, Alcalá de Henares (Madrid) 28871, Spain; § Instituto de Investigación Química “Andrés M. del Río” (IQAR), Universidad de Alcalá, Alcalá de Henares (Madrid) 28871, Spain

## Abstract

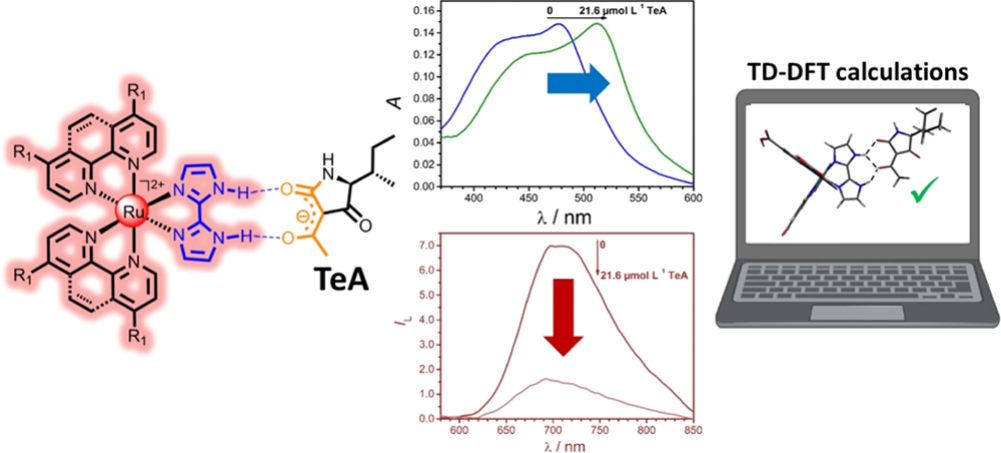

A family of ruthenium(II) complexes containing one 2,2′-biimidazole (bim) ligand and two polypyridyl (NN) ligands has been prepared and their photophysical and photochemical features have been tested in the presence of tenuazonic acid (TeA), a widespread food and feed mycotoxin of current concern. While not tested in in vivo studies, TeA and other secondary metabolites of *Alternaria* fungi are suspected to exert adverse effects on the human health, so sensors and rapid analytical procedures are required. It is well-known that 1,3-dicarbonyl compounds such as TeA are relatively easy to deprotonate (the p*K*_a_ of TeA is 3.5), yielding an enolate anion stabilized by resonance. The chelating and hydrogen-donor features of bim allow simultaneous binding to the metal core and to the target β-diketonate delocalized anion. Such a binding induces changes in the blue absorption (40 nm bathochromic shift), red luminescence intensity (>75% quenching), and triplet lifetime (0.2 μs decrease) of the Ru(NN)_2_(bim)^2+^ luminophore. Moreover, we have computationally rationalized, by time-dependent density functional theory, the structure of the different adducts of Ru–bim complexes with TeA and the electronic nature of the spectral absorption bands and their change upon the addition of TeA.

## Abstract 2

Luminescent heteroleptic Ru(II) complexes with one 2,2′-biimidazole ligand effectively bind 1,3-dicarbonyl enolates in polar organic media. A thorough experimental and computational study sheds light on the geometry and proton-transfer quenching of the hydrogen-bonded adduct unexplored so far but with applications in chemical sensing.

## Introduction

**Scheme 1:**
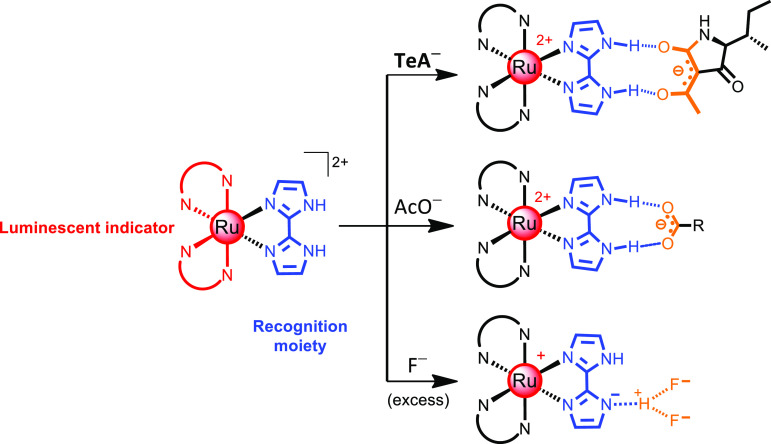
Suggested Interaction of Ru(II)–Bim Complexes with Various Anions in Non-protogenic Media: Tenuazonate (TeA^–^), Acetate (AcO^–^), and Fluoride (F^–^)

Anion recognition for optical sensing has drawn attention over the last years due to its critical role in chemical, biological, and environmental systems.^1^ Anions are essential for the interaction between proteins, the regulation of key cell metabolites, and the formation of enzyme–substrate complexes.^2^ Moreover, environmental monitoring of nitrates and phosphates that are used as fertilizers of crops is crucial to prevent eutrophication.^3^ The huge structural variety in terms of shape and dimensions of the anions represents a formidable challenge to the receptor design. Despite the scarcity of studies on β-diketonate anions, they have come out as key intermediates for the synthesis of anticancer drugs,^4^ chemical sensor receptors,^5^ food additives (e.g., curcumin),^6^ and bioactive species.^7^ Moreover, some of the 1,3-dicarbonyl compounds are noxious; for instance, tenuazonic acid (TeA, whose conjugated base is depicted in Scheme 1) and cyclopiazonic acid (CPA) are natural mycotoxins produced by *Alternaria* or *Penicillium* fungi that contaminate a large amount of the world’s food such as cereals, oilseeds, fruits, and vegetables.^8^ Out of all the *Alternaria* mycotoxins, TeA has been identified as the most dangerous one, showing both cytotoxic and phytotoxic effects.^9^

Among all the chemosensing platforms, luminescence-based sensors have been widely used for environmental monitoring, food safety assurance, and process analysis due to their high sensitivity, good selectivity, ease of miniaturization, and robustness, if luminescence lifetime rather than intensity measurements are carried out.^10,11^ In this regard, luminescent ruthenium(II) polypyridyl complexes are particularly suitable for chemical sensing due to their large Stokes shift (>150 nm), good thermal and photochemical stability, and their relatively long-lived excited state (up to 7 μs).^12^ Furthermore, their (photo)chemical and (photo)physical properties may be finely tuned by a judicious selection of the polyazaheterocyclic chelating ligands around the metal core. To report the presence of the TeA mycotoxin, a specific ligand must be introduced in the Ru(II) complex, in such a way that its interaction with the target analyte induces a change in the spectroscopic features of the complex. Due to the intrinsic acidity of TeA (p*K*_a_ = 3.5),^13^ a variety of tenuazonate (TeA^–^) forms may be present in solution arising from its double keto-enol tautomerism, the predominant tautomer being the one represented in Scheme 1.^13^ Its β-diketonate structure allows TeA^–^ to efficiently chelate metal cations.^14^ Moreover, its delocalized charge enables TeA^–^ to form hydrogen bonds and undergo electrostatic interactions with suitable partners.

Considering its chemical structure, we have recently proposed the 2,2′-biimidazole (bim) ligand as a suitable receptor for TeA.^15^ A double coordination feature allows bim to chelate a metal atom while keeping its ability to establish two simultaneous hydrogen bonds with different oxoanions of appropriate geometry (Scheme 1). Incorporation of bim to the coordination sphere of the metal complex considerably increases the acidity of the imidazole NH groups (p*K*_a_ of free H_2_bim, 11.5; p*K*_a1_ of [Ru(bpy)_2_(bim)]^2+^, 7.2).^16^ This enables strong hydrogen bonding or even deprotonation of bim in the presence of basic anions. It is not easy to clearly distinguish these two processes as several factors must be considered such as the acidity of the NH groups in the Ru(II) complex (it can be modulated through incorporation of electron-withdrawing or electron-donating groups in the ancillary ligands), the basicity of the interacting anion, the solvent, and the number and strength of the hydrogen bonds formed.^16^ Moreover, most of the investigated luminescent indicator dyes bear a positive charge; therefore, a significant electrostatic contribution must be considered in their interaction with negatively charged species.

Computational methods have demonstrated to be powerful tools to understand the factors that control the photophysical and photochemical features of Ru(II)–polypyridyl complexes.^17^ A few studies on the experimental recognition of halide, acetate (AcO^–^), phosphates, sulfates, and nitrates by Ru(II)–bim complexes have been reported.^16,18^ However, to the best of our knowledge, this is the first experimental and computational study of the interaction of these luminescent dyes with 1,3-dicarbonyl compounds.

Herein, we report the synthesis of a family of heteroleptic Ru(II) polypyridyl complexes containing a 2,2′-biimidazole ligand and a thorough investigation of their interaction with TeA by UV–vis and luminescence spectroscopies to optimize the optical sensing of this and related mycotoxins. The photophysical features of the Ru(II) dyes and their binding constants to TeA have been experimentally determined and computationally studied at the time-dependent density functional theory (TD-DFT) level. The latter enlightens the spectroscopic assignment and allows proposition of the most stable interaction geometry. Furthermore, the interaction with TeA has been compared to the binding to other monodentate (F^–^) and bidentate (AcO^–^) anions in organic non-protogenic media (Scheme 1).

## Experimental Section

### Chemicals

The precursor disodium 2,2′-bipyridine-4,4′-disulfonate (s2b), 2,2′-bipyridine-4,4′-diyldimethyl diacrylate (dab), and 2,2′-biimidazole (bim) ligands were synthesized by following reported procedures.^19−21^ The precursor *cis*-[Ru(NN)_2_Cl_2_] complexes were prepared according to the general method with minimal modifications.^22^ The “synthetic” tenuazonic acid, a mixture of 5*S*/5*R* (81:19) diastereoisomers, was obtained by a literature procedure.^5^ [Ru(phen)_2_(bim)]^2+^ (phen stands for 1,10-phenanthroline) and [Ru(dab)_2_(bim)]^2+^ were synthesized by the following literature methods, and their NMR data were found to be coincident with those reported.^15,23^ 2,2′-Bipyridine-4,4′-dicarboxylic acid (dcb) and tetrabutylammonium (TBA) acetate were purchased from Alfa Aesar (Germany). Ruthenium(III) chloride trihydrate, lithium chloride, and 2,2,6-trimethyl-4H-1,3-dioxin-4-one were from Acros Organics. l-isoleucine methyl ester hydrochloride, TBA hydroxide, TBA fluoride (*x*H_2_O), hexafluorophosphoric acid, butylhydroxytoluene (BHT), and sodium methoxide were from Merck. Ammonium hexafluorophosphate was purchased from Fluorochem. Sephadex LH20 was purchased from Cytiva. *m*-Xylene, anhydrous dimethyl sulfoxide (DMSO), and dimethyl formamide (DMF) were from Merck, whereas dichloromethane and methanol (all HPLC grade) were from VWR. Type I water was obtained with a Merck-Millipore Direct-Q3-UV system. Deuterated solvents with tetramethylsilane (TMS) as the internal reference were purchased from VWR.

### Instrumentation

UV–vis absorption spectra were recorded with a Varian Cary 3-Bio spectrophotometer (CA, USA). Steady-state emission spectra of the Ru(II) complexes in solution were measured with a FluoroSENS spectrofluorimeter (Gilden Photonics, UK) equipped with a red-sensitive Hamamatsu R928 photomultiplier and a 150-W xenon lamp. All the spectra have been corrected for the instrument response. Time-resolved emission spectra (TRES) and luminescence lifetimes of the indicator dyes in solution were measured with an Edinburgh Instruments (EI) FLS980-Xd2-T spectrometer, equipped with a Horiba NanoLED-470LH (463 nm, <1 ns pulse width), a 467 nm bandpass interference filter (Chroma), a 500 nm-blazed double monochromator in the emission channel, and a Hamamatsu R928P photomultiplier detector thermoelectrically cooled at −21 °C. The EI advanced fluorescence analysis software technology (FAST) was used to analyze the multiexponential decays and to perform the global analysis. ^1^H NMR spectra were obtained on Bruker Avance DPX 300 MHz-BACS60 and Bruker AV 500 MHz spectrometers; the latter was also used to record the ^13^C NMR spectra. Mass spectra (ESI) were measured with a Bruker HCT ultra spectrometer.

### Synthesis of [Ru(dcb)_2_(bim)]^2+^

A total of 250 mg (0.37 mmol) of *cis*-[Ru(dcb)_2_Cl_2_] and 50 mg of 2,2′-biimidazole (0.37 mmol) were dissolved in 3 mL of ethylene glycol. The solution was refluxed for 1 h under argon until the TLC analysis (silica; MeCN–water–KNO_3_ satd. aq 50:2:1 v/v/v) showed complete consumption of the starting materials. The reaction mixture was cooled, and 2 mL of water was added. The Ru(II) complex salt precipitated upon the addition of a few drops of saturated aqueous ammonium hexafluorophosphate was collected by vacuum filtration and washed with water. The resulting product was purified by dissolving the complex in 1 mmol L^–1^ NaOH solution and passing the solution through a Sephadex LH20 column. The adsorbed violet complex was eluted with methanol, and the fractions containing the sought product were combined and evaporated under reduced pressure. To remove the residual base, the product was dissolved in water and hexafluorophosphoric acid was added until acidic pH. The precipitated PF_6_^–^ salt of the complex was extracted into dichloromethane. The organic layer was separated, concentrated under reduced pressure, and dried under vacuum to yield the final pure product as a purple-reddish solid in 27% yield. ^1^H NMR (CD_3_CN, δ): 8.96 (d, *J* = 7.6 Hz, 4H_δ_), 8.10 (d, *J* = 5.9 Hz, 2H_α’_), 7.95 (d, *J*_1_ = 5.9 Hz, 2H_α_), 7.93 (d, *J*_1_ = 5.9 Hz, 2H_β’_), 7.74 (d, *J* = 5.9 Hz, 2H_β_), 7.30 (s, 2H_5_), 6.45 (s, 2H_4_). ^13^C NMR (DMSO-*d*_6_, δ): 165.1, 158.6, 158.3, 157.4, 153.2, 152.4, 139.3, 138.6, 128.1, 126.1, 123.3, 123.1 121.8, 117.1, 114.8. MS(ESI^–^) *m*/*z*: [M – H]^+^ calcd for C_30_H_19_N_8_O_8_Ru, 721.0; found, 720.8.

### Synthesis of [Ru(s2b)_2_(bim)]^2–^

A total of 200 mg (0.20 mmol) of *cis*-[Ru(s2b)_2_Cl_2_] and 30 mg of 2,2′-biimidazole (0.22 mmol) were dissolved in 9 mL of ethylene glycol. The solution was refluxed for 5 h under argon. The reaction mixture was cooled, and the Ru(II) complex salt precipitated upon the addition of acetone. The Ru(II) complex was collected by vacuum filtration and purified by re-precipitation in the acetone–diethyl ether mixture to yield the final pure product as a red solid in 70% yield. ^1^H NMR (DMSO-*d*_6_, δ): 8.49 (s, 4H_δ_), 7.97 (d, *J* = 5.8 Hz, 2H_α’_), 7.89 (d, *J* = 5.8 Hz, 2H_α_), 7.66 (dd, *J*_1_ = 5.8 Hz, *J*_2_ = 1.5 Hz, 2H_β’_), 7.55 (dd, *J*_1_ = 5.8 Hz, *J*_2_ = 1.5 Hz, 2H_β_), 7.38 (s, 2H_5_), 6.39 (s, 2H_4_). ^13^C NMR (DMSO-*d*_6_, δ): 157.4, 156.8, 155.3, 152.8, 151.7, 139.4, 127.5, 123.9, 123.3, 121.9, 119.4, 119.2. MS(ESI^–^) *m*/*z*: [M]^2–^ calcd for C_26_H_18_N_8_O_12_RuS_4_, 431.9; found, 431.7.

### Computational Details

All calculations were performed by applying DFT to the electronic ground state, including its TD-DFT when calculating the electronic excited-state properties. In particular, the B3LYP^24,25^ functional was used together with the Lanl2dz basis set to describe the metal atom and the 6-31 + G* basis set to describe all other atoms. The D3 version of the Grimme’s empirical dispersion with Becke-Johnson damping was included (D3(BJ)).^26,27^ The solvent (dimethyl sulfoxide, DMSO) was implicitly taken into account through the polarizable continuum model (PCM^28^) using the integral equation formalism variant (IEF-PCM). The Gaussian 16 suite of programs^29^ was used for these calculations.

## Results and Discussion

### Synthesis of the Luminescent Indicator Dyes

**Figure 1:**
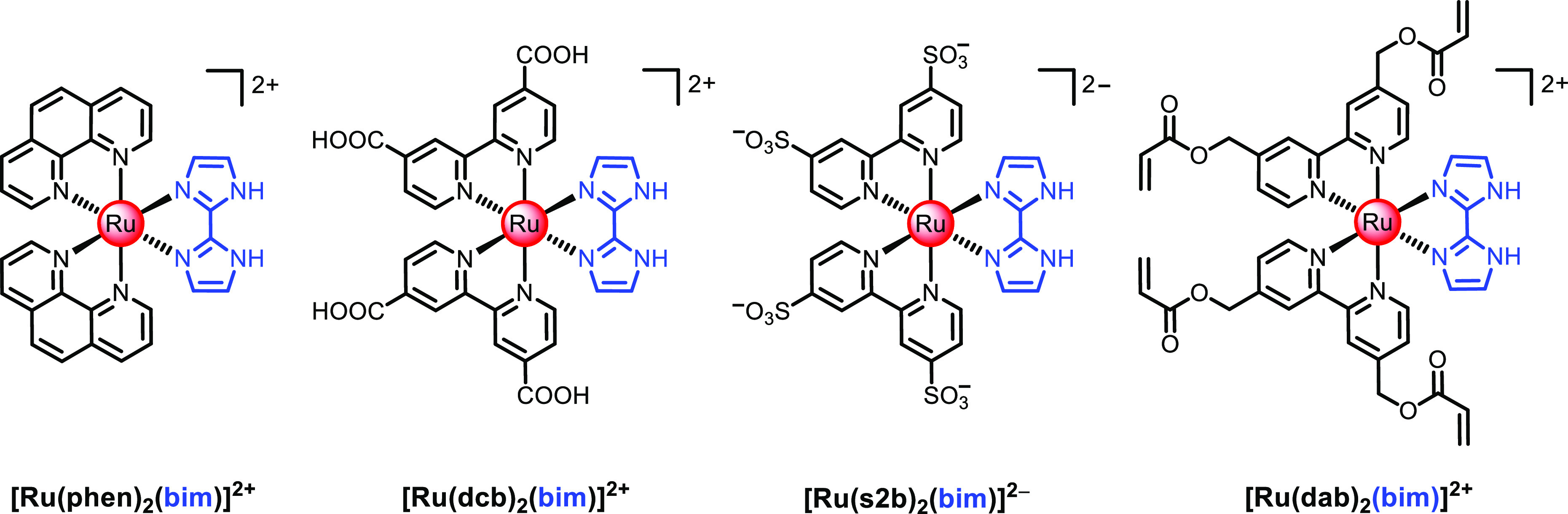
Chemical structure of the luminescent indicator dyes prepared in this work.

To explore the ability to bind 1,3-dicarbonyl species, a family of heteroleptic Ru(II)–polypyridyl complexes containing one 2,2′-biimidazole (bim) as the recognition moiety and two ancillary ligands has been prepared (Figure 1). The functional groups of these ancillary ligands (2,2′-bipyridine-4,4′-dicarboxylic acid, dcb; 2,2′-bipyridine-4,4′-disulfonate, s2b; and 2,2′-bipyridin-4,4′-diyldimethyl diacrylate, dab) have been chosen to eventually be able to tether the luminescent complexes to a polymeric matrix for chemical sensing applications (via carboxamide, sulfonamide, or radical polymerization).^15^ For the sake of comparison, the 1,10-phenanthroline (phen) complex was also prepared. The luminescent dyes were synthesized in a two-step route. First, the *cis*-Ru(NN)_2_Cl_2_ precursors were obtained according to the established procedure for the synthesis of *cis*-Ru(bpy)_2_Cl_2_,^22^ in which the chelating ligand was reacted with RuCl_3_ in the presence of a large excess of lithium chloride to prevent the formation of the tris complex. The target Ru(II) heteroleptic complexes were synthesized by refluxing the *cis*-Ru(NN)_2_Cl_2_ and bim. Due to the poor solubility of the latter, ethylene glycol was required. Figures S1–S9 in the Supporting Information show the structural confirmation of the synthesized Ru(II)–bim complexes by ^1^H NMR, ^13^C NMR, and ESI-MS.

### Spectroscopic Characterization of the Luminescent Probes

The absorption and photochemical features in DMSO of the prepared Ru(II) dyes are depicted in Table 1. These data were extracted from their corresponding absorption and emission spectra (Figure S10, Supporting Information). All the complexes show a broad absorption band in the blue which is assigned to an allowed d−π* metal-to-ligand charge transfer (MLCT) transition in agreement with the usual feature of Ru(II) polypyridyls and our computational results (Figure S11). In every case, the MLCT transition of the Ru–bim complexes in the visible absorption band exclusively involves the π* orbital of the bpy or phen ligands (Figure S11). This is a consequence of the much higher energy of the bim ligand π* orbital due to its electron-rich character. The high-energy region of the spectra is dominated by narrow intense absorption bands assigned to π–π* ligand-centered (LC) transitions of the bim and bpy (or phen) ligands.

**Table 1:** Absorption Data, Emission Maxima, and Luminescence Lifetimes of the Ru(II) Dyes in Air-Equilibrated DMSO Solutions at 25 ± 0.1 °C

Ru(II) dye	λ_abs_^max^/nm (ε/10^3^ mol L^–^^1^ cm^–^^1^)^a^	λ_em_^max^/nm^a^	Φ_em_^b^	τ_1_/ns^d^ (*B*_1_)	τ_2_/ns^d^ (*B*_2_)	τ_m_/ns^e^
**[Ru(phen)**_**2**_**(bim)]**^**2+**^	266 (82.1), 478 (11.3)	650	0.011	80 (1421)	177 (8430)	163
**[Ru(dcb)**_**2**_**(bim)]**^**2+**^	314 (48.0), 378 (14.3), 510 (15.0)	710	0.008	62 (1504)	153 (3990)	128
**[Ru(dab)**_**2**_**(bim)]**^**2+**^	292 (51.1), 341 (10.5), 490 (7.7)	701	0.003^c^	50 (322)	200 (247)	115
**[Ru(s2b)**_**2**_**(bim)]**^**2–**^	298 (56.9), 350 (13.2), 494 (11.2)	683	0.014	149 (3599)	503 (396)	184

a Peak wavelength uncertainty: ± 1 nm and molar absorption coefficient uncertainty: ± 5%.

b Luminescence quantum yields (sd ± 2%); measured in triplicate upon excitation at 475 nm, at (25 ± 0.1) °C and atmospheric pressure of 714 mm Hg; and reference: [Ru(bpy)_3_]Cl_2_, Φ_em_ = (0.040 ± 0.002) in H_2_O.^33^

c This value is somewhat underestimated due to the lack of response of the detector above 850 nm.

d Under air, the luminescence decays are fitted to the eq  (*i* = 2); goodness-of-the-fit indicator: χ^2^ ≤ 1.2; and uncertainties of the individual lifetimes: ± 2%.

e Pre-exponentially weighted average luminescence lifetime:  (*i* = 2); uncertainty: ± 1%.

Upon excitation, a relatively long-lived red luminescence is observed from the lowest-lying ^3^MLCT state (Table 1), originated by a fast intersystem crossing from the initially populated ^1^MLCT state. The bim ligand decreases the *t*_2g_^π^ (metal) → π*(ligand) back-donation, increasing the energy of the metal *t*_2g_^π^ orbitals.^30^ The latter shrinks the HOMO–LUMO energy gap, causing a red shift of the absorption and emission maxima compared to the corresponding homoleptic complexes [Ru(NN)_3_]^2+^ (454/617 nm for NN = bpy and 450/601 nm for NN = phen in DMSO, data not shown). In this way, the emission quantum yields and luminescence lifetimes of the Ru–bim complexes decrease. While [Ru(bpy)_3_]^2+^ displays an emission quantum yield of 0.045 and a lifetime around 0.7 μs in aerated acetonitrile,^31^ the Ru–bim complexes show emission quantum yields around 0.01 and emission lifetimes lower than 0.2 μs (Table 1). This fact might be ascribed to the smaller energy gap between the excited and ground states discussed above.^32^

### Interaction of Ru(II)–Bim Complexes with F^–^, AcO^–^, and TeA^–^

**Figure 2:**
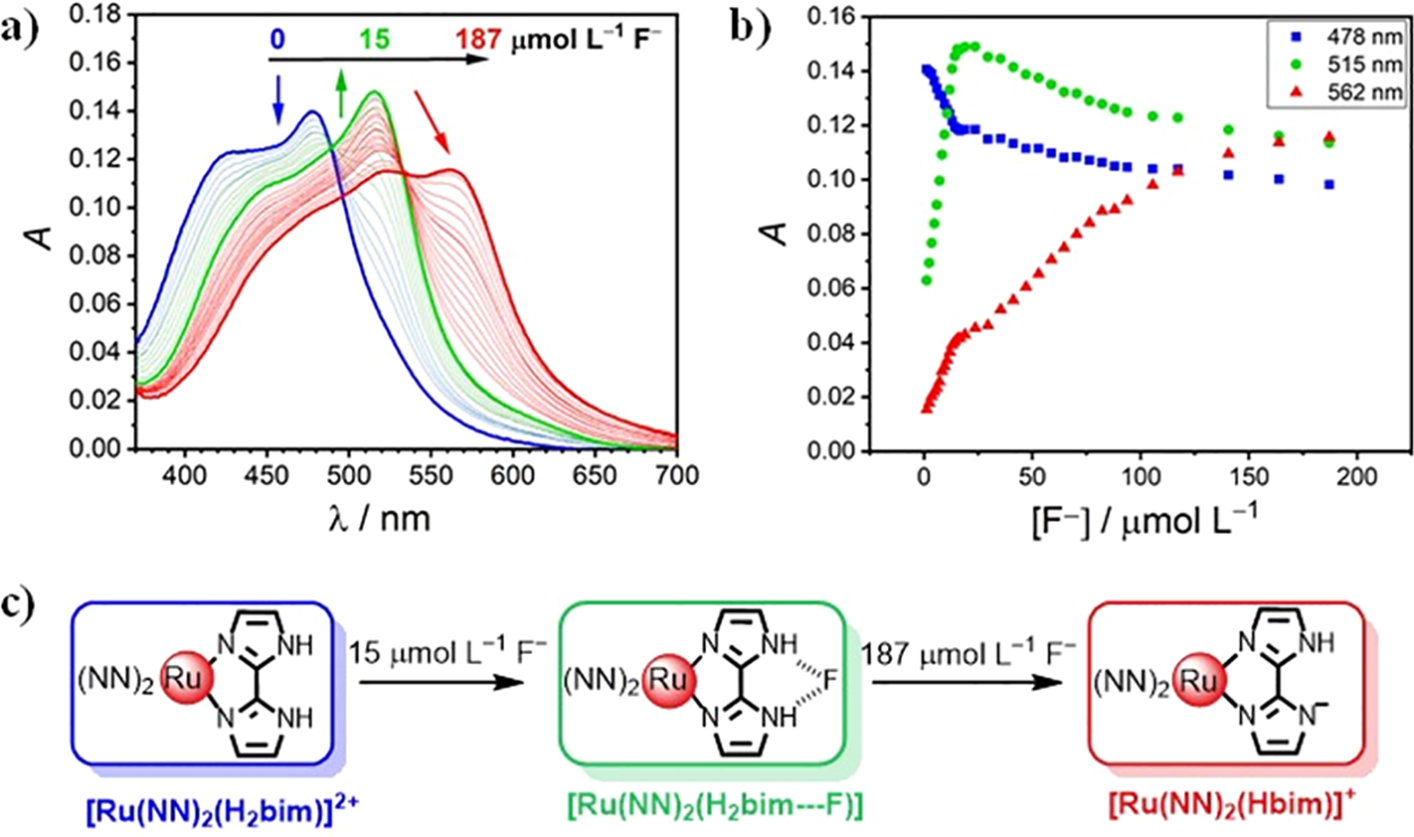
(a) Changes in the UV–vis absorption spectrum for the [Ru(phen)_2_(bim)]^2+^ complex (12.0 μmol L^–1^) in DMSO solution upon the addition of increasing amounts of F^–^. (b) Absorbance readings at 478, 515, and 562 nm vs the [F^–^] (μmol L^–1^). (c) Proposed species formed upon successive addition of F^–^.

The use of Ru(II)–bim complexes as metalloreceptors for luminescent sensing of various anions via hydrogen bonding has been reported.^18^ In these studies, fluoride (F^–^) is used to understand deprotonation of the Ru(II)–bim complexes in organic solvents due to the strong basicity of F^–^ in such media. Figure S12 (Supporting Information) depicts the absorption spectra of our luminescent complexes in the presence of increasing amounts of F^–^ (as TBA salt) in DMSO. As a case in point, Figure 2 shows that the addition of 1.25 mol F^–^ per mole of [Ru(phen)_2_(bim)]^2+^ induces a 37 nm bathochromic shift of its MLCT absorption band, with an isosbestic point at 489 nm. However, upon the addition of a large excess of F^–^, the absorption maximum at 515 nm gradually decreases, while a new band at 562 nm emerges (Figure 2). A new isosbestic point at 540 nm appears, indicating the direct formation of another species that might be the singly deprotonated complex (if the first equilibrium corresponds to an association) or the doubly deprotonated complex (if the first equilibrium corresponds to a deprotonation). These changes are fully reversible by acidification of the solutions and are visible to the naked eye (Figure S13): the initial yellow solution becomes orange and finally violet upon the addition of increasing amounts of F^–^. A similar behavior has been reported by Cui et al.^34^ for [Ru(bpy)_2_(bim)]^2+^ in acetonitrile solution.

To elucidate the exact nature of the involved species, a ^1^H NMR titration with F^–^ was carried out in DMSO-*d*_6_ (Figure S14, Supporting Information). The bim N–H signals should provide information on the interaction of [Ru(phen)_2_(bim)]^2+^ with the anion but, unfortunately, those protons were not observed in the spectrum. Nevertheless, the addition of an equimolar amount of F^–^ induces a broadening and upfield shift of 0.26 and 0.31 ppm of the bim H_4_ and H_5_ signals, respectively (Figure S14). Interestingly, when an excess of F^–^ is added, a new triplet at 16.5 ppm arises which can be attributed to the formation of the highly stable HF_2_^–^ species.^35,36^ The integral of this signal is compatible with the single deprotonation of the Ru(II) complex and only occurs when an excess of F^–^ is present in solution. Moreover, the H_4_ and H_5_ protons are further shifted upfield (0.29 and 0.38 ppm, respectively), suggesting an increase in the charge on the bim ligand from that observed in the presence of a stoichiometric amount of F^–^.

**Figure 3:**
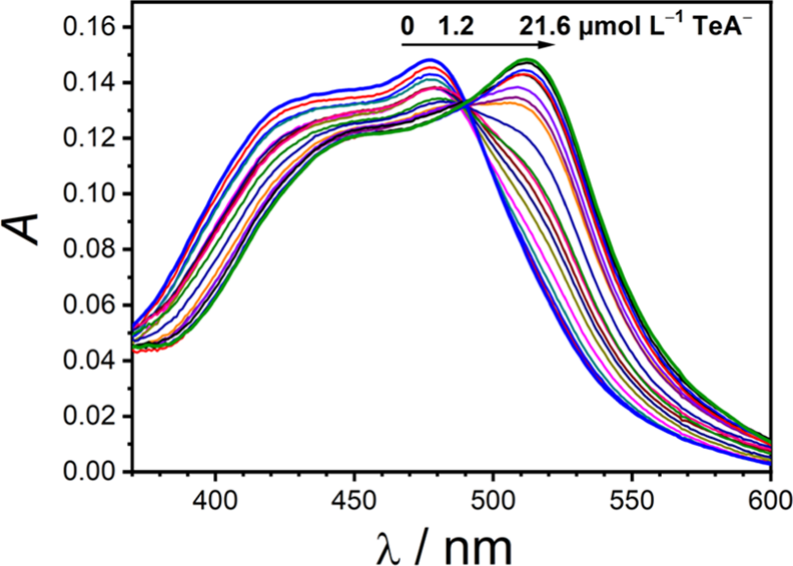
Changes in the UV–vis absorption of [Ru(phen)_2_(bim)]^2+^ (12.0 μmol L^–1^) in DMSO upon the addition of increasing amounts of TeA^–^ (as TBA salt).

To investigate whether or not TeA also influences the absorption features of the Ru(II)–bim complexes, we monitored their UV–vis absorption in the presence of tenuazonate in DMSO. As an example, Figure 3 shows that the addition of increasing amounts of the TeA toxin (as TBA salt) induces a shift from 478 to 512 nm in the absorption maximum of [Ru(phen)_2_(bim)]^2+^, with a well-defined isosbestic point at 490 nm. The latter is indicative of a 1:1 stoichiometry for the Ru(II)–tenuazonate adduct. For the sake of comparison, the same experiment was performed in the presence of AcO^–^ with similar results (the same changes were observed for all the Ru(II) complexes studied in the presence of AcO^–^ or TeA^–^, as shown in Figures S15 and S16, respectively). No further changes were detected when a large excess of AcO^–^ or TeA^–^ was added.

**Figure 4:**
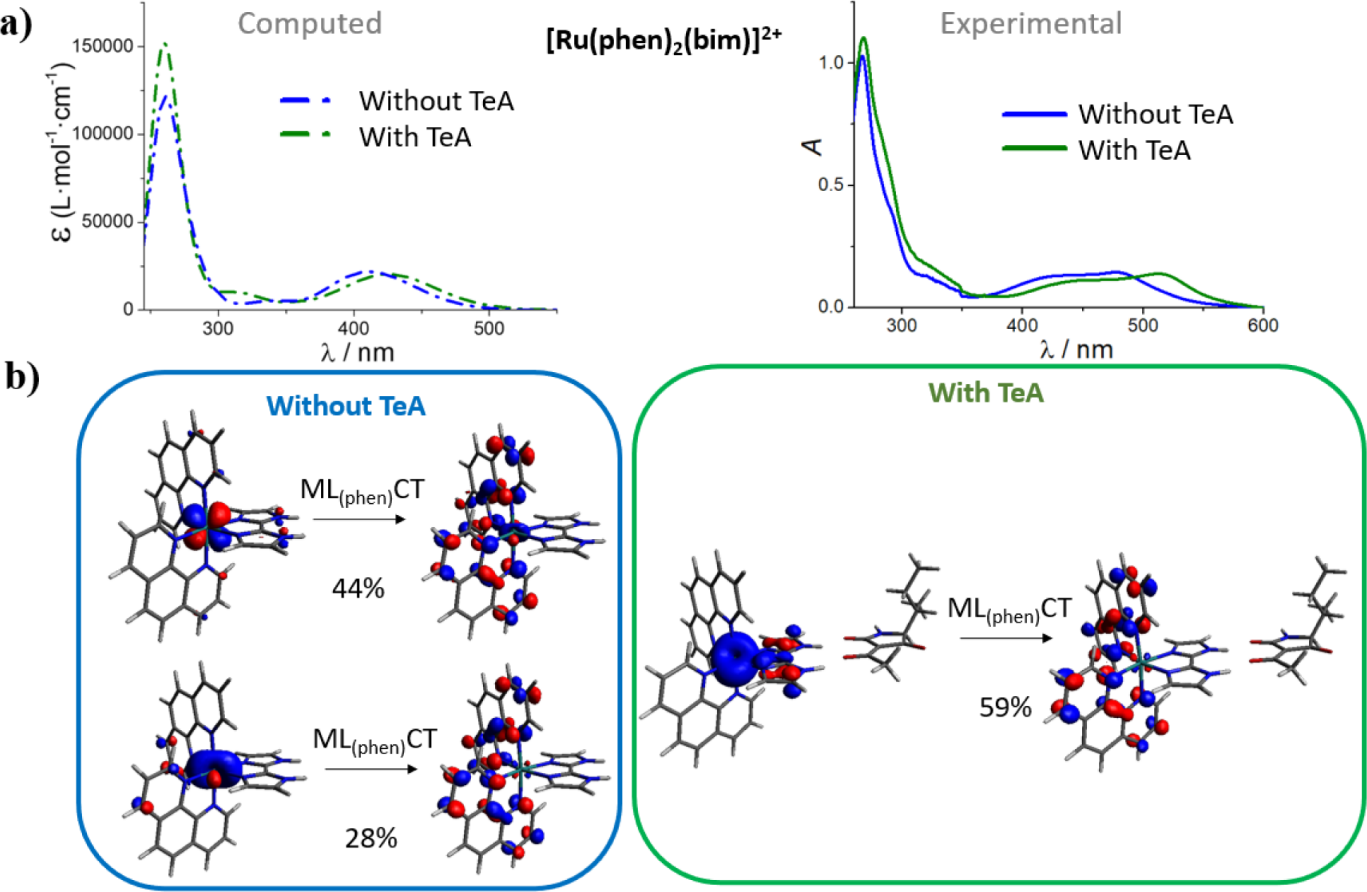
(a) Calculated vs experimental absorption spectrum of [Ru(phen)_2_(bim)]^2+^, in the absence (blue lines) or in the presence (green lines) of a hydrogen-bonded TeA^–^ molecule. (b) Molecular orbitals involved in the lowest-lying electronic transition of the visible absorption band, corresponding to a metal-to-phenanthroline charge transfer transition. Calculations have been performed with the ground-state-optimized (i.e., Franck–Condon) geometry (Table S2).

To confirm that the experimentally observed shifts of the visible absorption band of the Ru–bim complexes upon the addition of TeA^–^ are caused by the hydrogen-bond interactions of TeA^–^ with the bim ligand, we have computed their absorption spectra in the absence and in the presence of TeA^–^ (Figures 4 and S16 in the Supporting Information). The latter confirms that, in both cases, the lowest energy band corresponds to a MLCT transition for all the luminescent indicator dyes under study. More specifically, our calculations indicate that the MLCT band arises from a charge transfer from the metal center to the bipyridine or phenanthroline ligands and not to the higher-lying π*(bim) orbital (see above). In agreement with the experimental results, we also found out that the hydrogen-bonded TeA^–^–bim interaction in DMSO produces a red shift in the calculated absorption spectra (Figures 4a and S16). Furthermore, it is computationally confirmed that the band centered at ca. 300 nm is not sensitive to the addition of TeA^–^ because it encompasses several ligand-centered transitions mainly involving the π orbitals of the substituted bipyridines or phenanthroline.

### Geometry of the Association of TeA^–^ with the Ru–Bim Complexes

**Figure 5:**
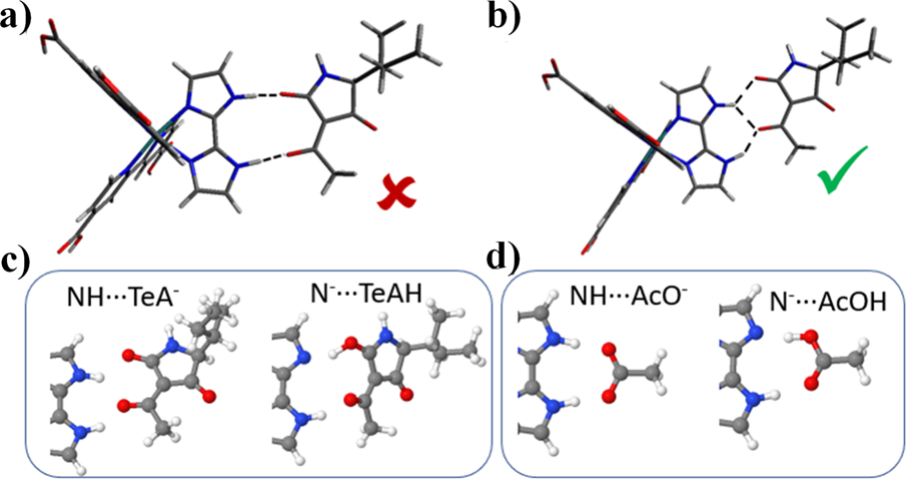
(a) Initially proposed geometry (in DMSO) of the [Ru(dcb)_2_(bim)]^2+^–TeA^–^ adduct with the two C–O groups of TeA^–^ in front of the bim N–H moieties. Starting from this unstable geometry, various stable structures characterized by a rearrangement of the NH···O^δ−^ intermolecular interactions were found computationally (Figure S18). (b) Calculated most stable geometry. (c) Geometry of the most stable [Ru(dcb)_2_(bim)]^2+^–TeA^–^ adduct and of the calculated N^–^···HO proton transfer adduct. (d) Geometry of the most stable [Ru(dcb)_2_(bim)]^2+^–AcO^–^ adduct and of the calculated N^–^···HO proton transfer adduct.

Once demonstrated the formation of [Ru(NN)_2_(bim)]–tenuazonate adducts through UV–vis absorption measurements, we investigated their geometry by computational methods. We have also compared the geometry of the TeA^–^ adduct with that reported for AcO^–^.^34^ First, we have modeled TeA^–^ to evaluate the negative charge delocalization over the two C–O groups in β relative position (lactam and acetyl) of its most stable conformation (Scheme 1 and Figure S17 in the Supporting Information). We have found that the charges on these two oxygen atoms involved in the interaction with bim are similar. Therefore, they should be equivalent for interacting with the NH moieties of bim. Based on this finding, our first proposal for calculations was an association of TeA^–^ in front of the NH moieties of bim, leading to two hydrogen bonds (Figure 5a). In fact, this structure is similar to that reported for AcO^–^,^34^ based on the X-ray structure of the adduct. Computationally, we have found this geometry to be stable for the bim–AcO^–^ adduct but not for the bim–TeA^–^ analogue. In contrast, two isoenergetic geometries have been computationally discovered for the bim–TeA^–^ adduct, characterized by the location of one of the C–O groups between the two NH moieties of the bim ligand (Figures 5b and S18). It should be underlined that, as expected, the computed properties (i.e., excitation energies) for these two isoenergetic geometries are almost identical. Several other geometries of the bim–TeA^–^ adduct have been explored and computationally optimized, but all of them are significantly less stable than the selected one (Figure S19). The alternative (similar) adduct of the keto and acetyl C–O groups of TeA^–^, also in β relative position, displays always a higher computed energy due to the steric crowding of the 2-butanoyl chain of TeA^–^ and the imidazole ligand.

Furthermore, we have computationally evaluated the possible proton transfer from bim to TeA^–^ or AcO^–^ in the ground-state adducts. To this aim, we have calculated the energy of the interacting species before (NH···TeA^–^, NH···AcO^–^) and after (N^–^···TeAH, N^–^···AcOH) the single proton transfer (Figure 5c,d). In both cases, the N^–^···HO species resulting from this proton transfer is less stable than the original situation. However, while this energy difference is ca. 3 kcal mol^–1^ for the bim–AcO^–^ adduct regardless of the ancillary ligands of Ru(II), the energy difference ranges from 9 to 24 kcal mol^–1^ for the bim–TeA^–^ adducts (Table S1 in the Supporting Information).

### Luminescence Quenching Upon TeA^–^ Binding

**Figure 6:**
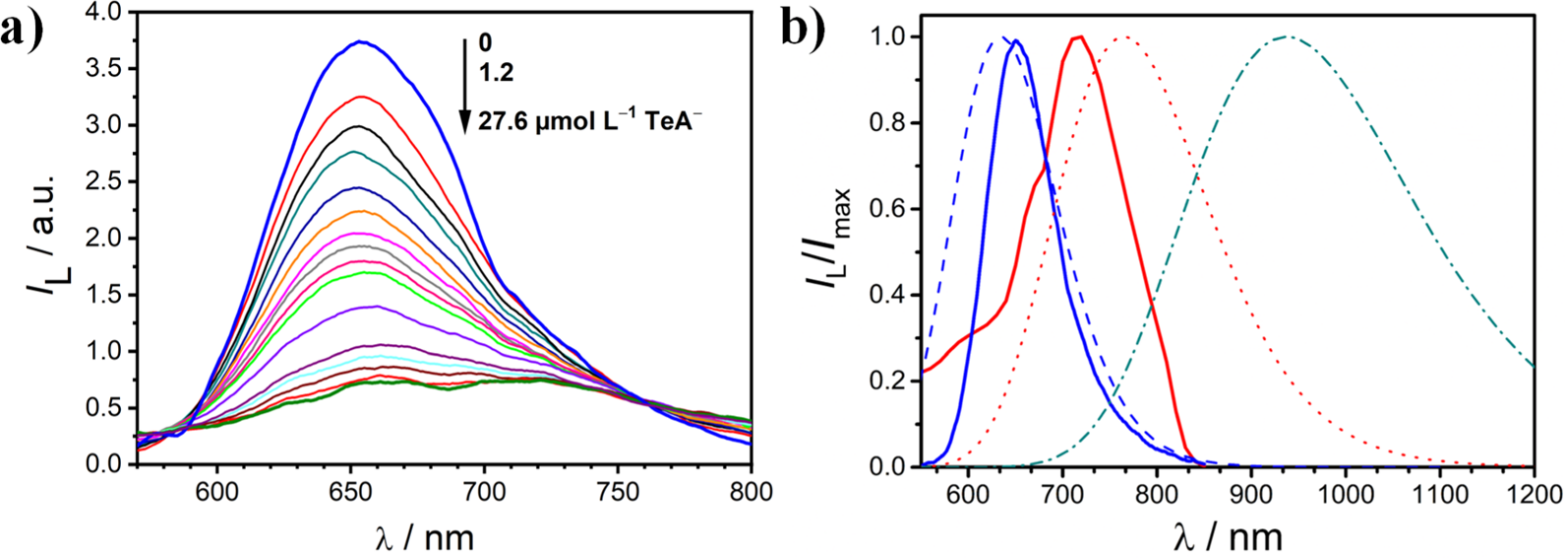
(a) Changes in the luminescence spectra of [Ru(phen)_2_(bim)]^2+^ in DMSO (λ_exc_ = 490 nm; 12.0 μmol L^–1^) upon the addition of increasing amounts of TeA^–^ (as TBA salt). (b) Time-resolved emission spectra (TRES, solid lines) of [Ru(phen)_2_(bim)]^2+^ (14.0 μmol L^–1^) in DMSO, in the presence of a stoichiometric amount of TeA^–^ upon laser excitation at 463 nm. The TRES were obtained by slicing and addition of the wavelength-dependent luminescence decays in the 160–180 ns (solid red line) and 300–700 ns (solid blue line) time windows, respectively. This figure includes the calculated emission spectra of [Ru(phen)_2_(bim)]^2+^ (dashed line) and its deprotonated (dotted line) and doubly deprotonated (dotted-dashed line) forms in DMSO.

To further understand the chemical behavior of the indicator dyes, the effect of TeA^–^ on the luminescence of the Ru–bim complexes was evaluated in DMSO. Upon excitation at 490 nm (absorption isosbestic point), [Ru(phen)_2_(bim)]^2+^ exhibits an emission maximum at 660 nm (Figure 6a). This luminescence is 82% quenched upon the addition of 2 mol TeA^–^ per mole of Ru complex, with gradual broadening. The latter suggests the presence of different luminescent species due to the hydrogen-bonded adduct formation. A similar response to TeA^–^ was observed in the emission spectra of the other Ru(II)–bim dyes (Figure S20 in the Supporting Information).

Additionally, we studied the consequences of the TeA^–^ binding on the luminescence of the Ru(II)–bim complexes by time-resolved detection in aerated DMSO (Figure S21 in the Supporting Information). In all cases, the luminescence decay in the absence of TeA^–^ requires the sum of two exponentials to successfully fit it (Table 1). As a case in point, [Ru(phen)_2_(bim)]^2+^ shows lifetime components of 177 ns (93%) and 80 ns (7%) (). Due to traces of water in the spectroscopic-grade DMSO (<0.1%) and crystallization water molecules in the solid Ru(II) complexes (see Experimental Section), the least abundant component would correspond to a small amount of the singly deprotonated species. This phenomenon has already been observed for the luminescence of [Ru(dab)_2_(bim)]^2+^ in acetonitrile–water medium.^15^ Therefore, an irreversible proton transfer or a slow proton exchange and not an acid–base equilibrium is taking place in their ^3^MLCT excited state (otherwise, a single exponential would be observed). An irreversible deprotonation in the excited state has indeed been observed for a 2-pyridylimidazole Ru(II) complex in water.^37^ For the sake of comparison, we also calculated the so-called “pre-exponentially weighted” mean lifetime (τ_m_, Table 1) of each decay profile, a robust parameter that characterizes multiexponential decays.^38^

A global analysis of the luminescence lifetimes of [Ru(phen)_2_(bim)]^2+^ in the presence of increasing amounts of TeA^–^ in DMSO is shown in Table S3 (Supporting Information). The global analysis requires two components for a satisfactory fit (χ_global_ = 1.08). This result suggests that the fully protonated and singly deprotonated excited species would be responsible for the luminescence observed under the former conditions, while the doubly deprotonated excited species is not observed as a consequence of its strongly red-shifted position (see below). A final 25% decrease in the mean luminescence lifetime (from 150 to 112 ns) was observed upon the addition of TeA^–^. If we compare the degree of quenching measured by steady-state luminescence (Figure 6a) and that estimated from the mean emission lifetimes, we have to conclude that a variable degree of static quenching is occurring as it would be expected from the formation of a [Ru(phen)_2_(bim)]^2+^–TeA^–^ adduct. Pure static quenching has been observed for [Ru(phen)_2_(iip)]^2+^ complexes (iip = 2-imidazolyl-2-imidazo[4,5-f]phenanthroline) in the presence of Cu(II) due to the high association constant of the latter.^17^

**Figure 7:**
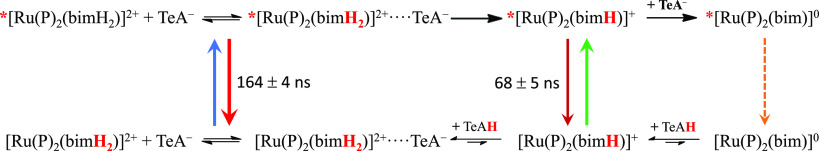
Schematic representation of the different processes that may occur in the presence of TeA^–^ in the ground and excited state of [Ru(phen)_2_(bim)]^2+^. Note that the fully protonated biimidazole ligand has been called “bim” throughout the text and not “bimH_2_”.

To investigate the exact nature of the excited species responsible for the emission components mentioned above, a TRES analysis of the photoexcited [Ru(phen)_2_(bim)]^2+^ was carried out in the presence of a stoichiometric amount of TeA^–^ in DMSO (Figure 6b). The shorter-lived luminescent species (68 ns) displays its emission maximum at 720 nm, while the luminescence of the longer-lived component (164 ns) peaks at 650 nm. This result agrees with the assignment of the short and long components to the singly deprotonated and fully protonated excited species we have made as mentioned above. The expected shorter lifetime of the deprotonated species is due to the energy-gap rule. Nevertheless, the emission maximum of the doubly deprotonated *[Ru(phen)_2_(bim)] species is not observed. This might be ascribed to its further red-shifted emission well into the NIR as suggested by the theoretical calculations (Figure 6b). The shift is a consequence of an additional increase in the π-donor character of the doubly deprotonated bim ligand which will destabilize the metal complex HOMO. A summary of the ground- and excited-state processes involving [Ru(phen)_2_(bim)]^2+^ in the presence of TeA^–^ is depicted in Figure 7. The photoacidity of our [Ru(NN)_2_(bim)]^2+^ complexes, species that are only able to undergo proton transfer to TeA in their excited state, is not without precedent; the acidity of the bipyridinedicarboxylic (dcb) ligand of [Ru(NN)_2_(dcb)]^2+^ (NN = electron-withdrawing bpy ligand) increases 1 order of magnitude upon excitation.^39^

### Association Constants of Ru(II)–Bim Complexes with TeA^–^

To determine the association constants (*K*_a_) of the Ru(II)–bim complexes to TeA^–^ from the spectral luminescence data in DMSO, the HypSpec program (v1.1.50, Protonic software, www.hyperquad.co.uk), based on the solution of the equations of mass balance by the Newton–Raphson method, was used.^40^ The resulting *K*_a_ values for the different [Ru(NN)_2_(bim)]^2+^–TeA^–^ adducts, assuming an 1:1 stoichiometry, have been collected in Table 2. These *K*_a_ values have also been computationally calculated by selecting the ground-state minimum depicted in Figure 5b. Specifically, the association Gibbs free energy has been computed as the difference between those of the reactants and of each [Ru(NN)_2_(bim)]^2+^–TeA^–^ adduct. For the sake of comparison with the computed data, the association Gibbs free energies were also calculated from the *K*_a_ values in Table 2 using the well-known van’t Hoff equation (Δ*G*_a_ = −*RT* ln *K*_a_). The computed values support the experimental data: [Ru(s2b)_2_(bim)]^2–^ displays the lowest association constant, whereas the *K*_a_ values for the other bim complexes are 150-fold larger and display similar values. The overall negative charge of the former complex or competition of its sulfonate groups would be the reason for its much lower *K*_a_ with TeA^–^ in DMSO.

**Table 2:** Association Constants Determined by Luminescence Spectroscopy and the Corresponding Experimental and Computed Gibbs Free Energies (Δ*G*_a_) of the [Ru(NN)_2_(bim)]^2+^–TeA^–^ Adducts in DMSO at (25 ± 1) °C

	experimental		computed	
Ru(II) complex	*K*_a_/10^5^ (M^–1^)	Δ*G*_a_ (kcal mol^–1^)	Δ*G*_a_ (kcal mol^–1^)	*K*_a_/10^3^ (M^–1^)
[Ru(s2b)_2_(bim)]^2^^–^	0.039	–4.9	–1.7	0.018
[Ru(phen)_2_(bim)]^2+^	6.3	–7.9	–4.5	2.0
[Ru(dcb)_2_(bim)]^2+^	6.2	–7.9	–5.2	6.5
[Ru(dab)_2_(bim)]^2+^	6.8	–8.0	–5.2	6.5

## Conclusions

Luminescent ruthenium(II) polypyridyl complexes containing one 2,2′-biimidazole (bim) ligand and two ancillary ligands for an eventual covalent binding to solid supports can be used as molecular probes for 1,3-dicarbonyl compound sensing in organic media. A thorough spectroscopic and computational study of a family of [Ru(NN)_2_(bim)]^2+^ complexes, in the presence of the conjugated base of the tenuazonic acid mycotoxin, has allowed us to discern the electronic and structural factors that control the hydrogen-bonding interaction between the luminescent probes and the 1,3-dicarbonyl compound. Our findings demonstrate the formation of a hydrogen-bonded adduct between the tenuazonate anion and the bim moiety rather than a single deprotonation. The latter is only accomplished when an excess of more basic anions such as fluoride is present in the organic solvent. Nevertheless, the higher acidity of the ^3^MLCT excited state of the [Ru(NN)_2_(bim)]^2+^ complexes, caused by localization of the photoexcited electron on the ancillary polypyridyl ligands (NN), leads to an irreversible proton transfer to tenuazonate with significant quenching of the Ru-complex luminescence. A comprehensive investigation of the luminescence lifetimes and time-resolved emission spectroscopy in the presence of tenuazonate evidences the Ru-complex photoacidity. Our work paves the way for the design of novel Ru(II)–bim complexes as intensity- and lifetime-based luminescent sensors of relevant β-dicarbonyl-containing analytes. More applications in this regard are currently being sought in our laboratories.

## Supporting Information Available

^1^H NMR, ^13^C NMR, and mass spectra of the luminescent indicator dyes, absorption and emission spectra of the Ru(II) dyes in DMSO, absorption and emission spectra of the Ru(II)–bim complexes in DMSO, electronic transitions involved in each absorption band, changes in the Ru(II) dyes upon the addition of F^–^, color changes in [Ru(phen)_2_(bim)]^2+^ upon the addition of F^–^, ^1^H NMR spectra of [Ru(phen)_2_(bim)]^2+^ after the addition of increasing amounts of F^–^, absorption changes in the Ru(II) dyes upon the addition of AcO^–^, computed and experimental UV–vis spectra of the Ru(II) complexes in the absence and in the presence of TeA^–^, structure optimization of TeA^–^, calculated possible ground-state geometries of the [Ru(phen)_2_(bim)]^2+^–TeA^–^ adduct, emission changes in the Ru(II) dyes upon the addition of TeA^–^, luminescence decays of the photoexcited Ru(II) complexes, schematic representation of the different processes that may occur in the presence of TeA^–^ in the ground and excited state, association energies between the Ru(II) complex and TeA^–^ or AcO^–^, global analysis of the luminescence lifetimes of [Ru(phen)_2_(bim)]^2+^ in the presence of TeA^–^, and Cartesian coordinates in Ångström of the ground-state DFT-optimized structures (PDF)
