# Supplementary material for: Interaction of a 1,3-Dicarbonyl Toxin with Ru(II)-Biimidazole Complexes for Luminescence Sensing: A Spectroscopic and Photochemical Experimental Study Rationalized by Time-Dependent Density Functional Theory Calculations
Source: Inorg Chem. 2021 Dec 19;61(1):328–37. doi: 10.1021/acs.inorgchem.1c02887 (PMC8753653; doi:10.1021/acs.inorgchem.1c02887)
Supplement: Supplementary file 1 — ic1c02887_si_001.pdf [file ic1c02887_si_001.pdf]

Supporting Information

INTERACTION OF A 1,3-DICARBONYL TOXIN WITH Ru(II)-BIIMIDAZOLE COMPLEXES FOR LUMINESCENCE SENSING: A SPECTROSCOPIC AND PHOTOCHEMICAL EXPERIMENTAL STUDY RATIONALIZED BY TD-DFT CALCULATIONS

José Quílez-Alburquerque,[†] *Cristina García-Iriepa,*[‡,§,]* *Marco Marazzi,*[‡,§] *Ana B. Descalzo,*[†] *Guillermo Orellana*[†,]*

[†] Department of Organic Chemistry, Faculty of Chemistry, Universidad Complutense de Madrid (UCM), 28040 Madrid, Spain.

[‡] Departamento de Química Analítica, Química Física e Ingeniería Química, Universidad de Alcalá, 28871 Alcalá de Henares (Madrid), Spain.

[§] Instituto de Investigación Química "Andrés M. del Río" (IQAR), Universidad de Alcalá, 28871 Alcalá de Henares (Madrid), Spain.

***Corresponding Authors:** E-mail address: orellana@quim.ucm.es (G. Orellana) cristina.garciai@uah.es (C. García-Iriepa)

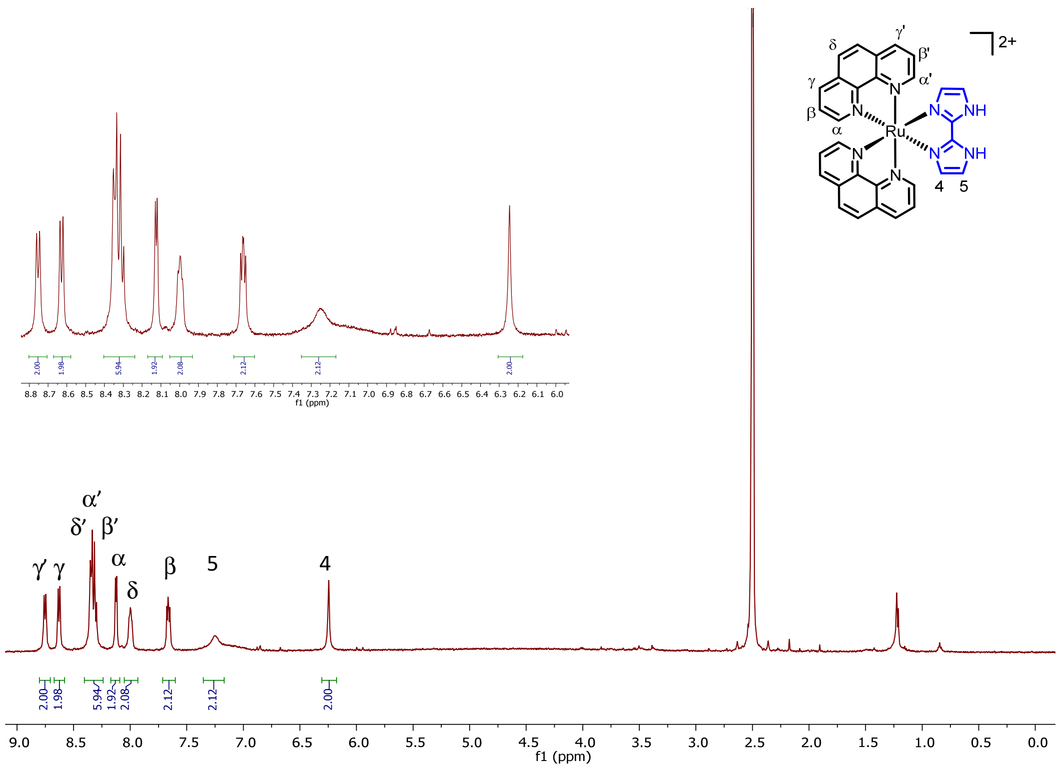

Figure S1. 500-MHz ^{1}H NMR spectrum of $[Ru(phen)_2(bim)]^{2+}$ in DMSO-d_6. The proton assignment of this and the other complexes was carried out according to Orellana et. al.[1] The inset shows the signals in the aromatic region.

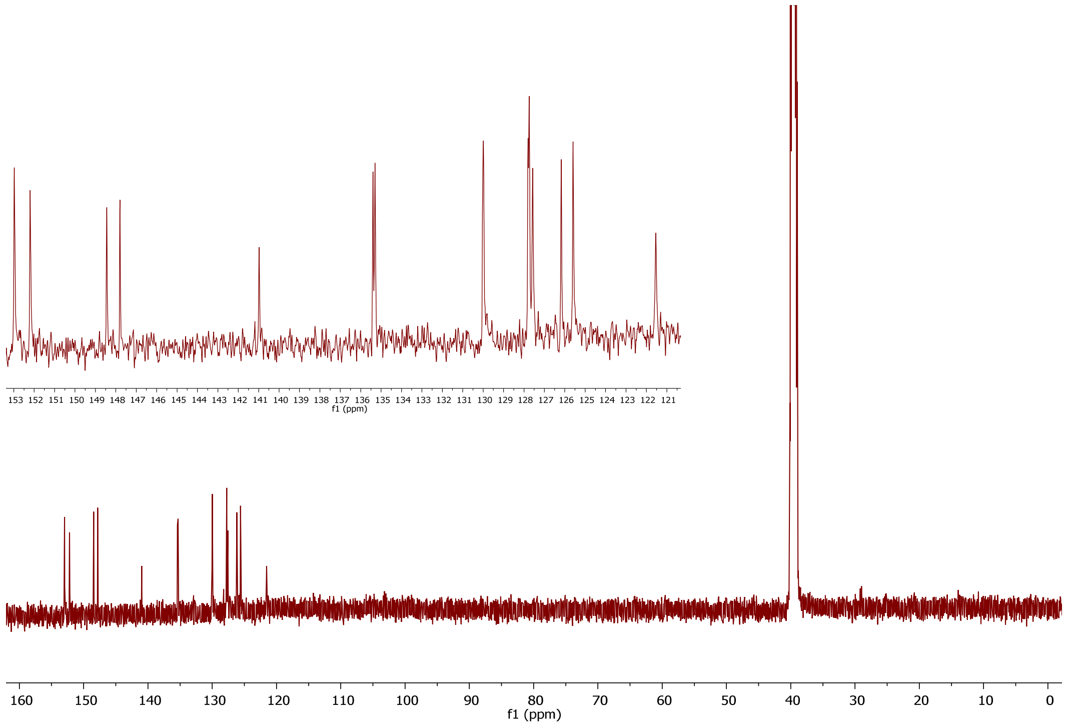

Figure S2. 125-MHz ^{13}C NMR spectrum of $[Ru(phen)_2(bim)]^{2+}$ in DMSO-d_6. The inset shows the signals in the aromatic region.

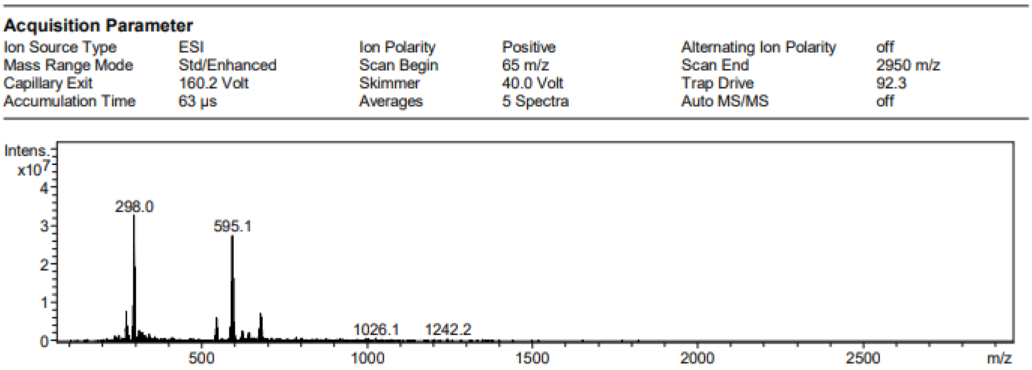

Figure S3. Mass spectrum of $[Ru(phen)_2(bim)]^{2+}$ recorded in MeOH by electrospray ionization in positive detection mode; m/z: [M] calcd. for $[C_{30}H_{21}N_8Ru]^+$ and $[C_{30}H_{22}N_8Ru]^{2+}$, 595.1; 298.0; found, 595.1; 298.0.

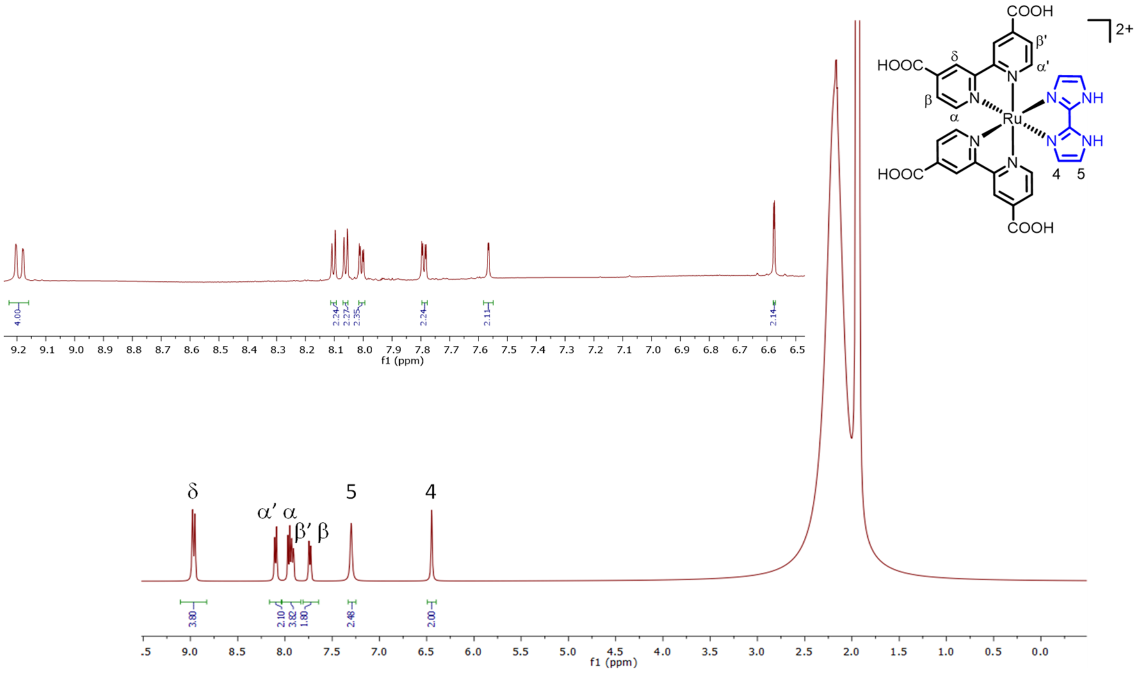

Figure S4. 300-MHz ^{1}H NMR spectrum of $[Ru(dcb)_2(bim)]^{2+}$ in acetonitrile-d_3. The inset shows the 500-MHz ^{1}H NMR spectrum (DMSO-d_6) of the aromatic region.

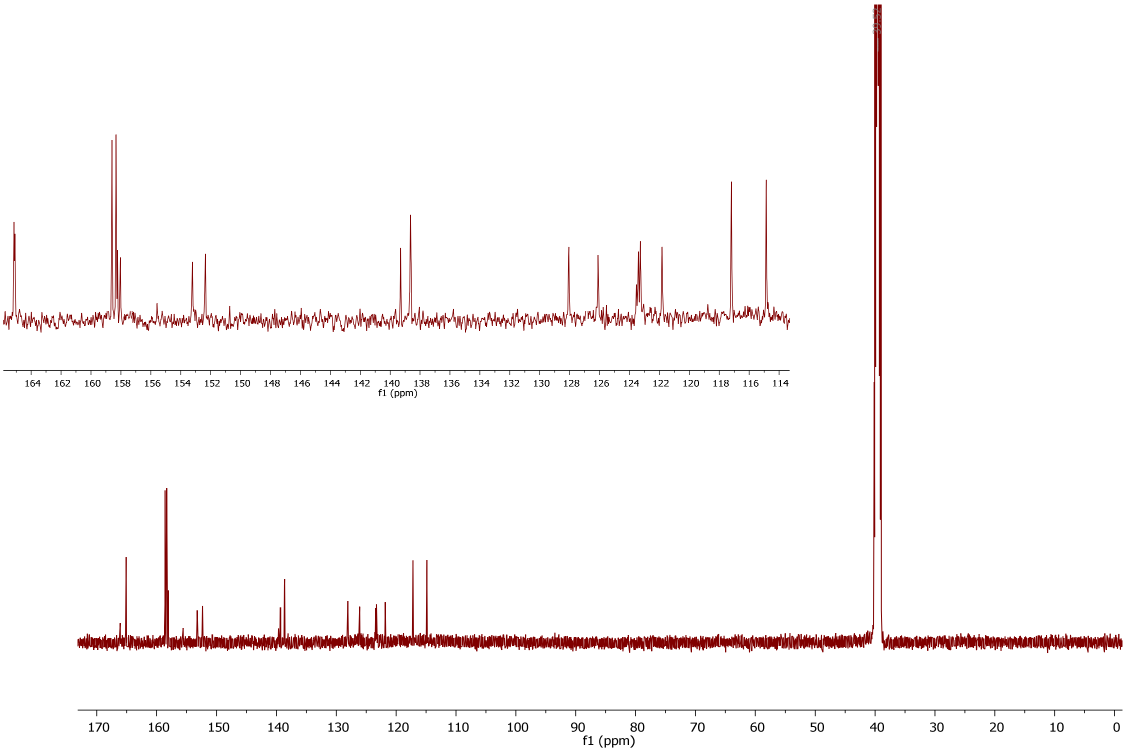

Figure S5. 125-MHz ^{13}C NMR spectrum of $[Ru(dcb)_2(bim)]^{2+}$ in DMSO-d_6. The inset shows the signals in the aromatic region.

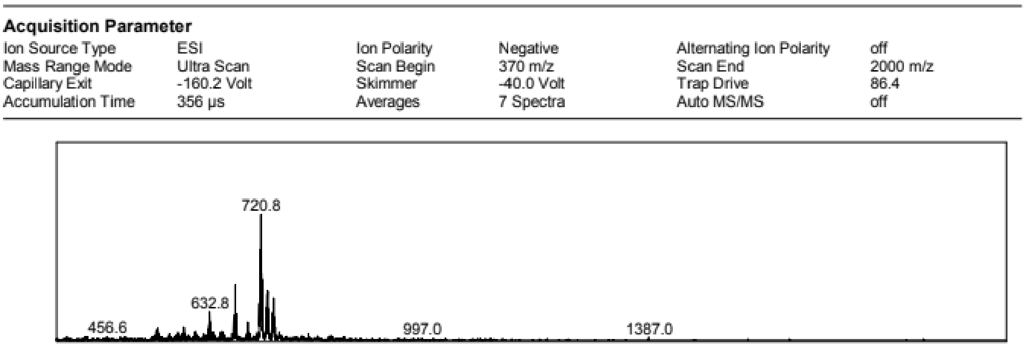

Figure S6. Mass spectrum of $[Ru(dcb)_2(bim)]^{2+}$ recorded in MeOH by electrospray ionization in negative detection mode; m/z: calcd. for $[C_{30}H_{19}N_8O_8Ru]^-$, 721.0; found, 720.8.

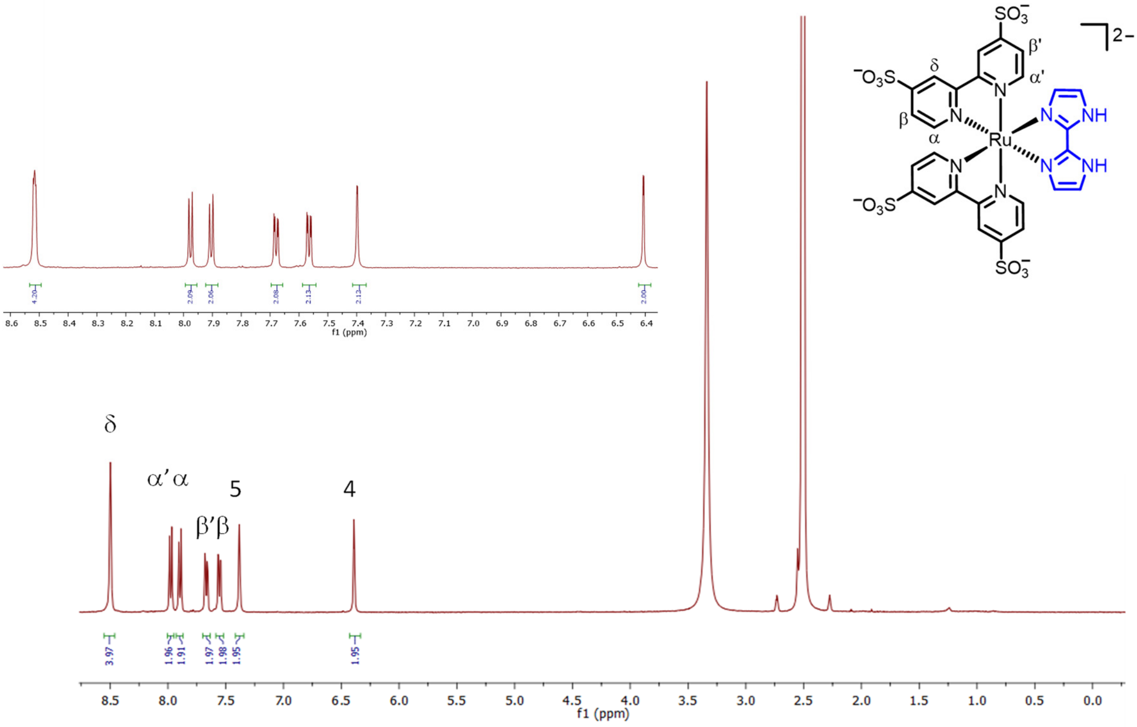

Figure S7. 300-MHz ^{1}H NMR spectrum of $[Ru(s2b)_2(bim)]^{2-}$ in DMSO-d_6. The inset shows the 500-MHz ^{1}H NMR spectrum of the aromatic region.

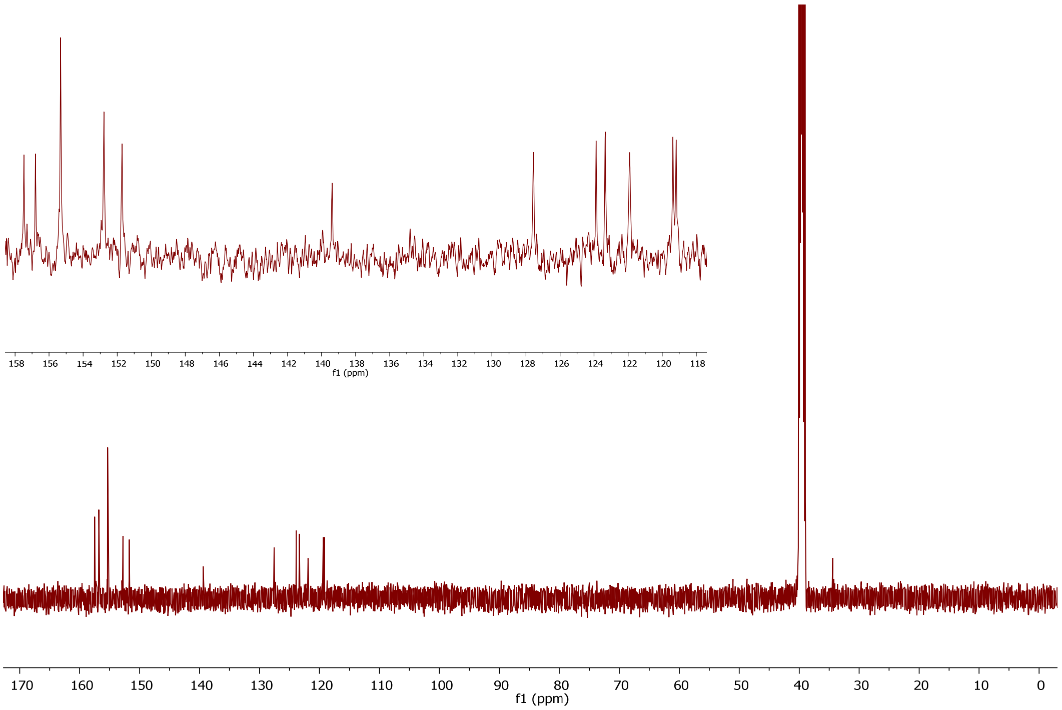

Figure S8. 125-MHz ^{13}C NMR spectrum of $[Ru(s2b)_2(bim)]^{2-}$ in DMSO-d_6. The inset shows the signals in the aromatic region.

Acquisition Parameter

Ion Source Type	ESI	Ion Polarity	Negative	Alternating Ion Polarity	off
Mass Range Mode	Ultra Scan	Scan Begin	370 m/z	Scan End	2000 m/z
Capillary Exit	-160.2 Volt	Skimmer	-40.0 Volt	Trap Drive	86.4
Accumulation Time	124 µs	Averages	7 Spectra	Auto MS/MS	off

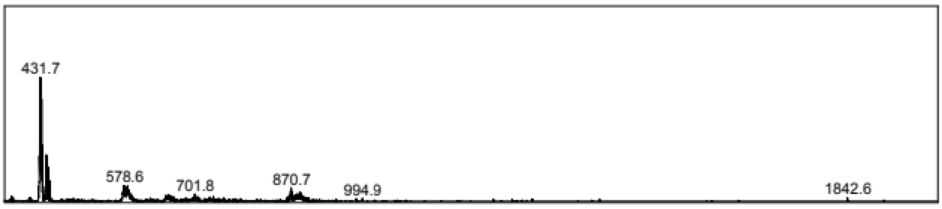

Figure S9. Mass spectrum of $[Ru(s2b)_2(bim)]^{2-}$ recorded in MeOH by electrospray ionization in negative detection mode; m/z: $[M]^{2-}$ calcd. for $[C_{26}H_{18}N_8O_{12}RuS_4]^{2-}$, 431.9; found, 431.7.

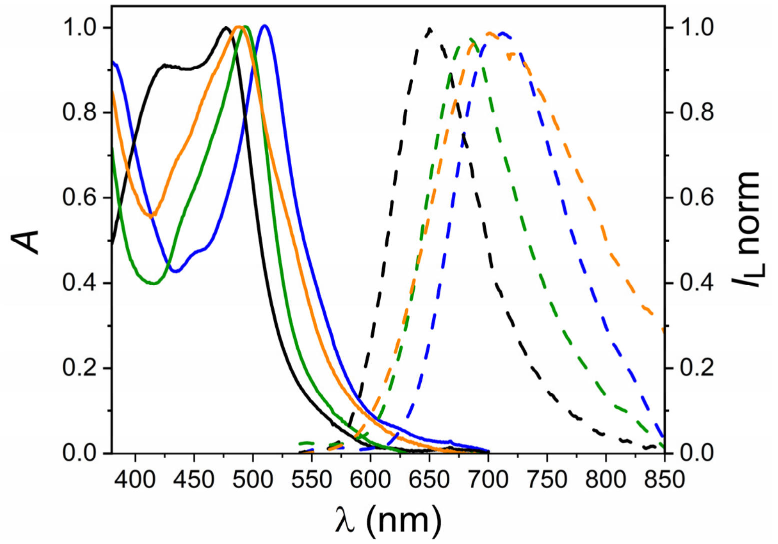

Figure S10. Absorption and emission spectra in DMSO of $[Ru(phen)_2(bim)]^{2+}$ (black solid and dashed line), $[Ru(dcb)_2(bim)]^{2+}$ (blue solid and dashed line), $[Ru(dab)_2(bim)]^{2+}$ (orange solid and dashed line) and $[Ru(s2b)_2(bim)]^{2-}$ (green solid and dashed line).

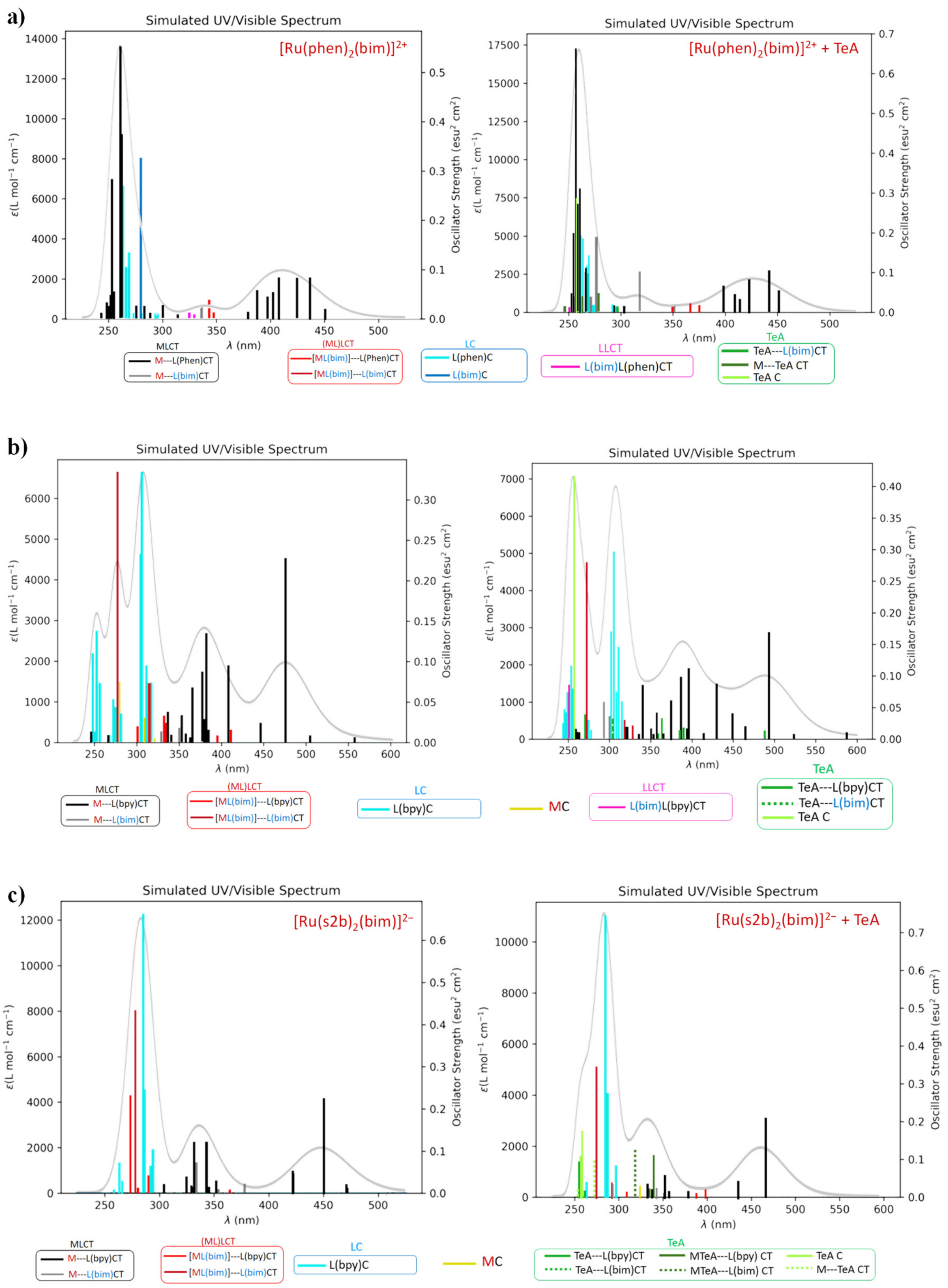
a)
Simulated UV/Visible Spectrum
[Ru(phen)2(bim)]2+
Simulated UV/Visible Spectrum
[Ru(phen)2(bim)]2+ + TeA
ε(L mol−1 cm−1)
Oscillator Strength (esu2 cm2)
λ (nm)
MLCT
M---L(Phen)CT
M---L(bim)CT
(ML)LCT
[ML(bim)]---L(Phen)CT
[ML(bim)]---L(bim)CT
LC
L(phen)C
L(bim)C
LLCT
L(bim)L(phen)CT
TeA
TeA---L(bim)CT
M---TeA CT
TeA C
b)
Simulated UV/Visible Spectrum
Simulated UV/Visible Spectrum
ε(L mol−1 cm−1)
Oscillator Strength (esu2 cm2)
λ (nm)
MLCT
M---L(bpy)CT
M---L(bim)CT
(ML)LCT
[ML(bim)]---L(bpy)CT
[ML(bim)]---L(bim)CT
LC
L(bpy)C
MC
LLCT
L(bim)L(bpy)CT
TeA
TeA---L(bpy)CT
TeA---L(bim)CT
TeA C
c)
Simulated UV/Visible Spectrum
[Ru(s2b)2(bim)]2−
Simulated UV/Visible Spectrum
[Ru(s2b)2(bim)]2− + TeA
ε(L mol−1 cm−1)
Oscillator Strength (esu2 cm2)
λ (nm)
MLCT
M---L(bpy)CT
M---L(bim)CT
(ML)LCT
[ML(bim)]---L(bpy)CT
[ML(bim)]---L(bim)CT
LC
L(bpy)C
MC
TeA
TeA---L(bpy)CT
TeA---L(bim)CT
MTeA---L(bpy) CT
MTeA---L(bim) CT
TeA C
M---TeA CT

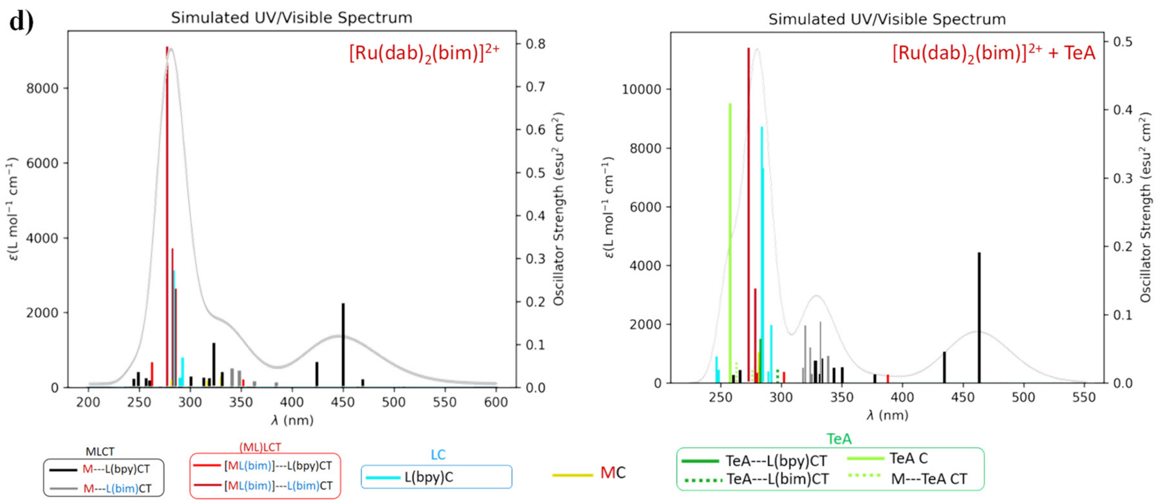

Figure S11. Calculated electronic absorption spectra in the absence and in the presence of TeA⁻ and electronic transitions involved in each absorption band of a) $[Ru(phen)_2(bim)]^{2+}$, b) $[Ru(dcb)_2(bim)]^{2+}$, c) $[Ru(s2b)_2(bim)]^{2-}$, and d) $[Ru(dab)_2(bim)]^{2+}$.

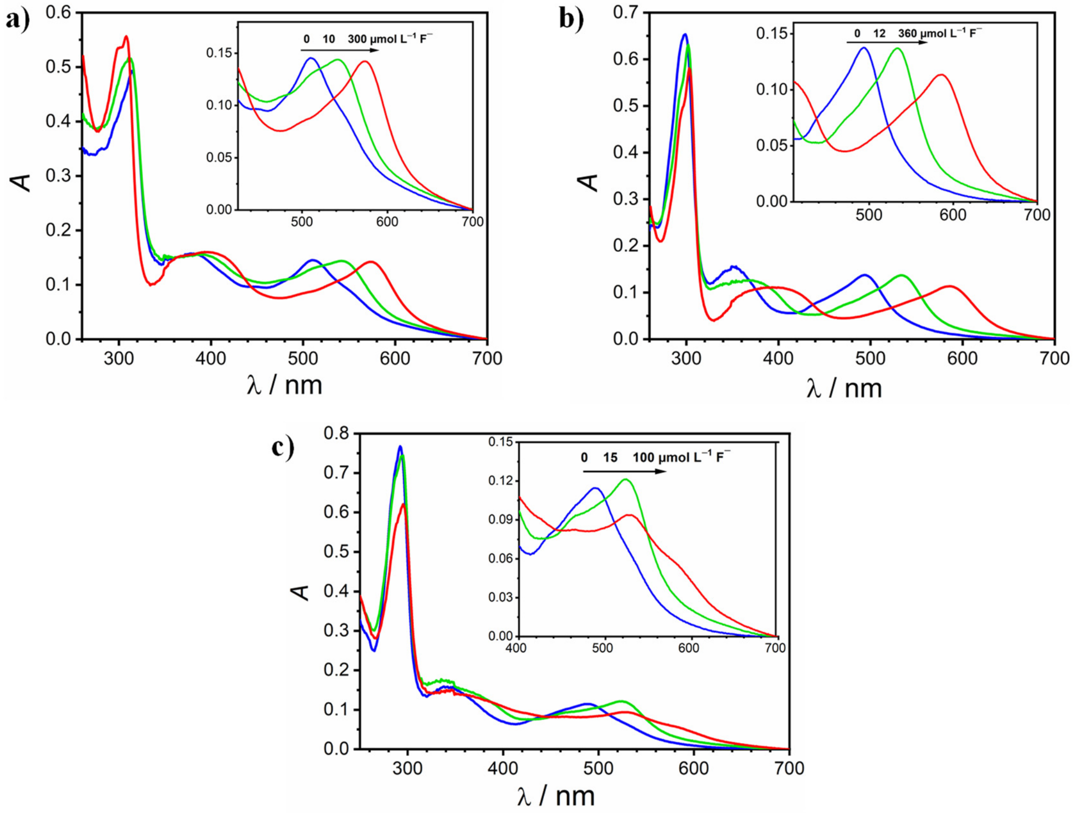

Figure S12. Changes in the UV-vis absorption for a) $[Ru(dcb)_2(bim)]^{2+}$ (12.0 µmol L^{-1}), b) $[Ru(s2b)_2(bim)]^{2-}$ (12.0 µmol L^{-1}), and c) $[Ru(dab)_2(bim)]^{2+}$ (15.0 µmol L^{-1}) in DMSO upon the addition of increasing amounts of F^- (as TBA salt).

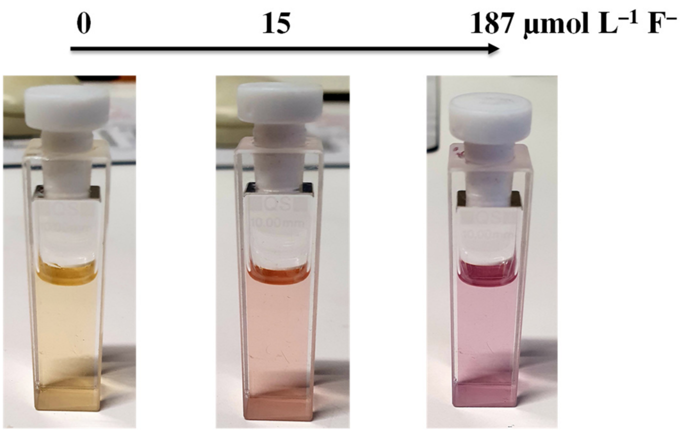

Figure S13. Color changes observed in DMSO for $[Ru(phen)_2(bim)]^{2+}$ (12.0 μmol L^{-1}) after addition of 15 and 187 μmol L^{-1} of F^-, respectively (as TBA salt).

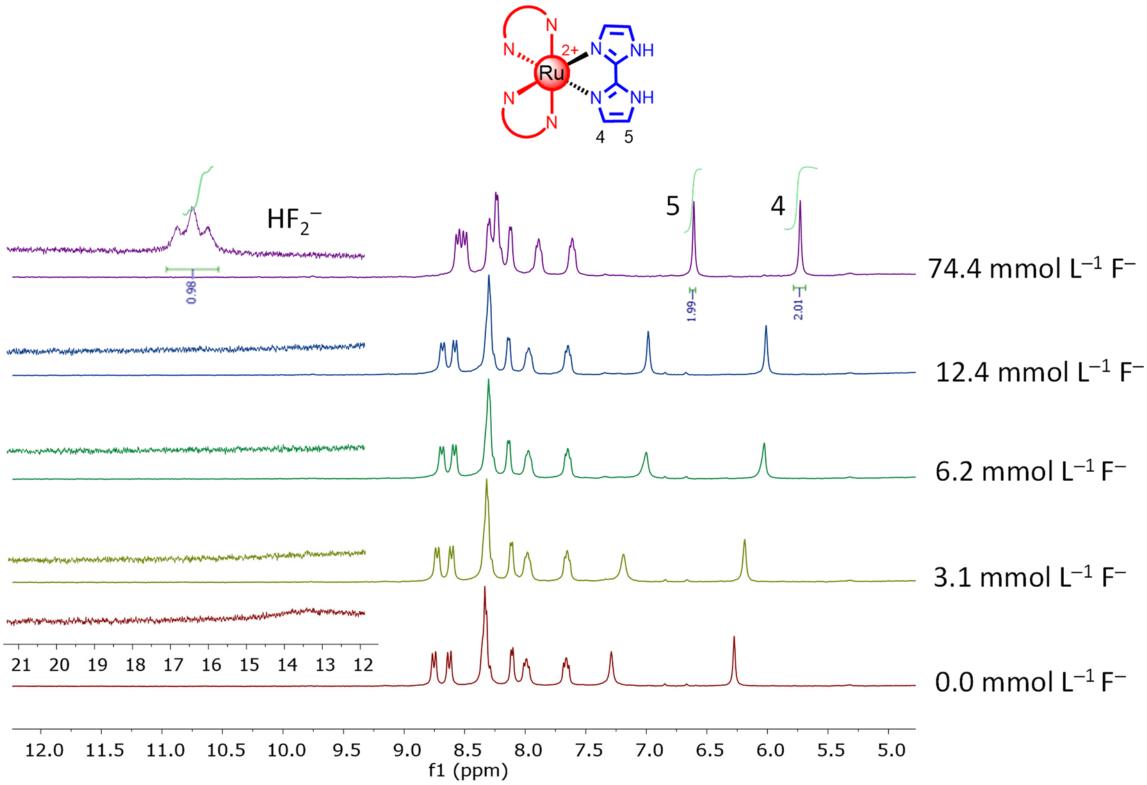

Figure S14. 300-MHz ^{1}H NMR spectrum of $[Ru(phen)_2(bim)]^{2+}$ (6.2 mmol L^{-1} in DMSO-d_6) after addition of increasing amounts of F^- (as TBA salt). The inset shows the formation of the HF_2^- species at high concentrations of fluoride, indicating deprotonation of the coordinated bim ligand.

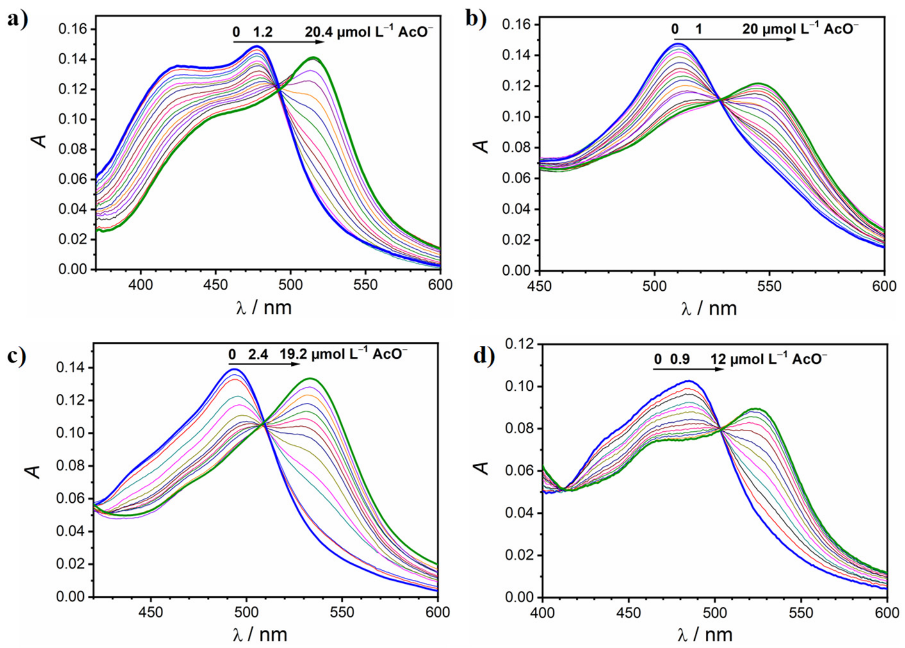

Figure S15. Changes in the UV-vis absorption for a) $[Ru(phen)_2(bim)]^{2+}$ (12.0 µmol L^{-1}), b) $[Ru(dcb)_2(bim)]^{2+}$ (10.0 µmol L^{-1}), c) $[Ru(s2b)_2(bim)]^{2-}$ (12.0 µmol L^{-1}), and d) $[Ru(dab)_2(bim)]^{2+}$ (9.0 µmol L^{-1}) in DMSO upon addition of increasing amounts of AcO^- (as TBA salt).

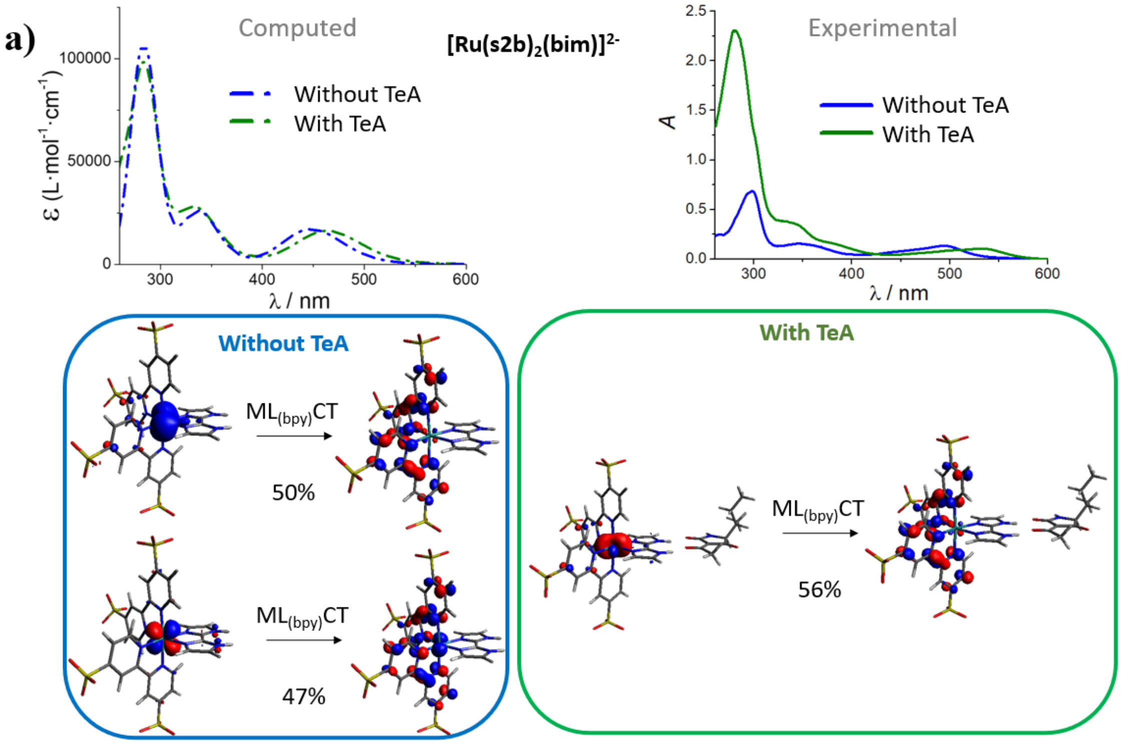

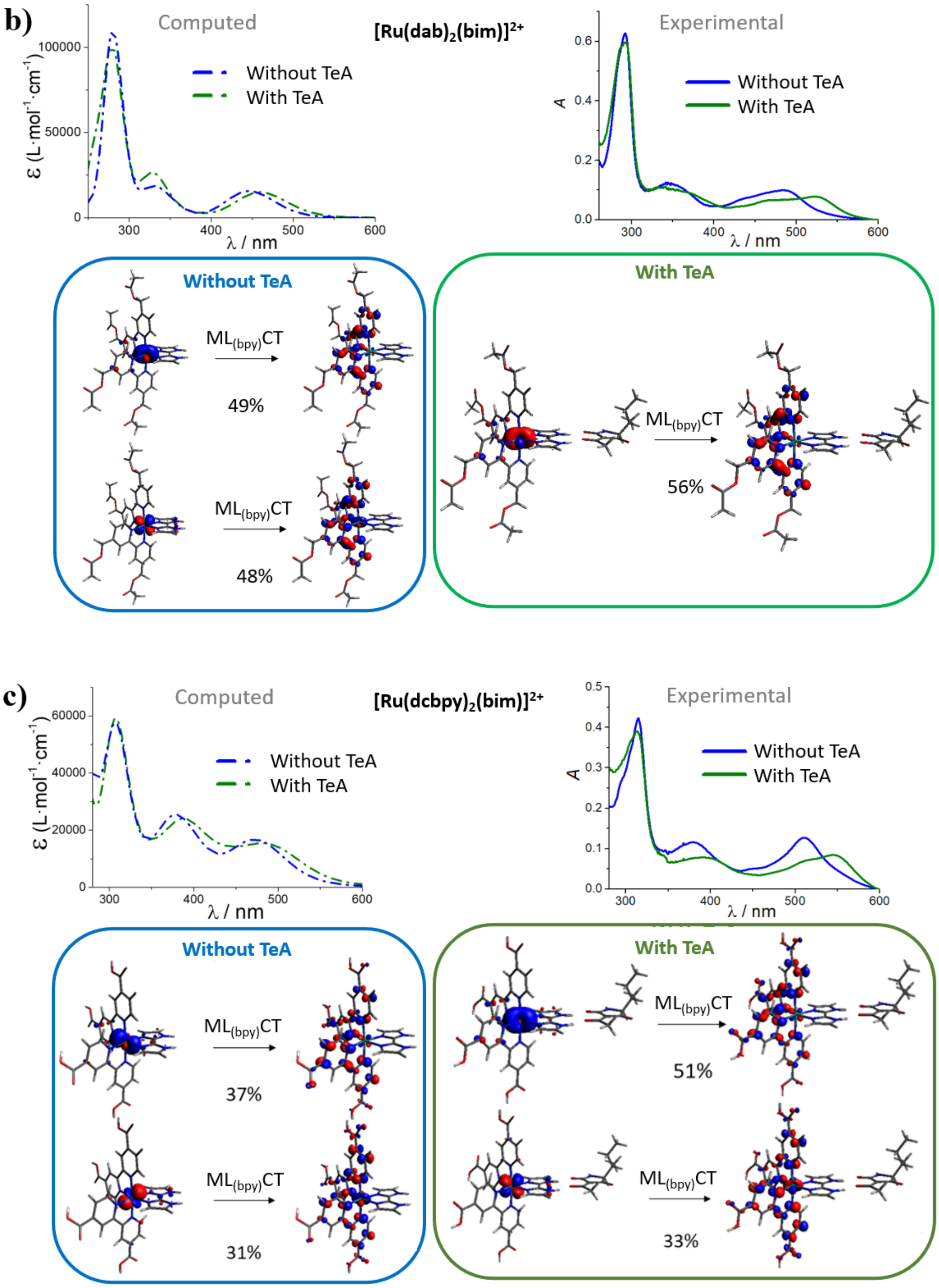

Figure S16. Calculated vs. experimental absorption spectra in the absence (blue lines) and in the presence (green lines) of TeA^-, and molecular orbitals involved in the electronic transition corresponding to the lowest energy band of a) $[Ru(s2b)_2(bim)]^{2-}$, b) $[Ru(dab)_2(bim)]^{2+}$, and c) $[Ru(dcb)_2(bim)]^{2+}$.

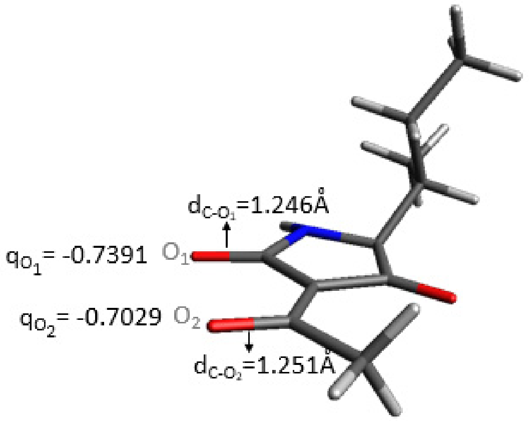

Figure S17. Structure of the ground state (S_0) minimum of TeA^-. The C–O distances and natural bond order (NBO) charges of the two oxygen atoms involved in the interaction with the Ru(II) complexes are depicted.

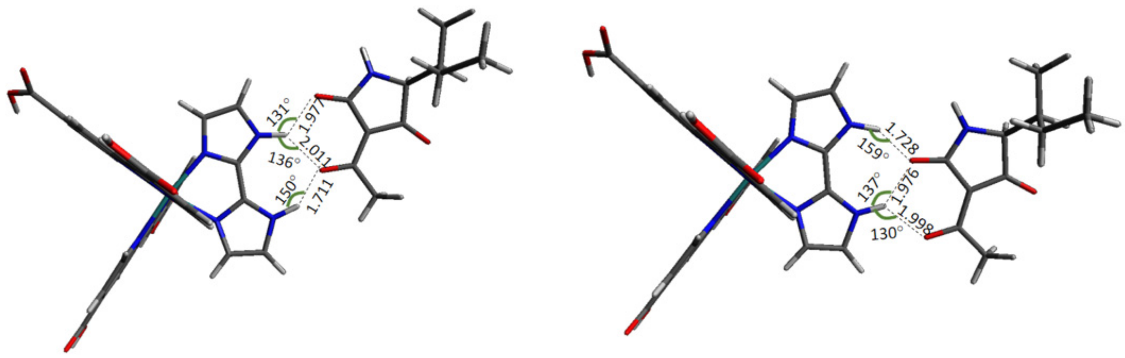

Figure S18. Structural parameters of the $[Ru(dcb)_2(bim)]^{2+}$–TeA^- adduct for the two similar most stable geometries.

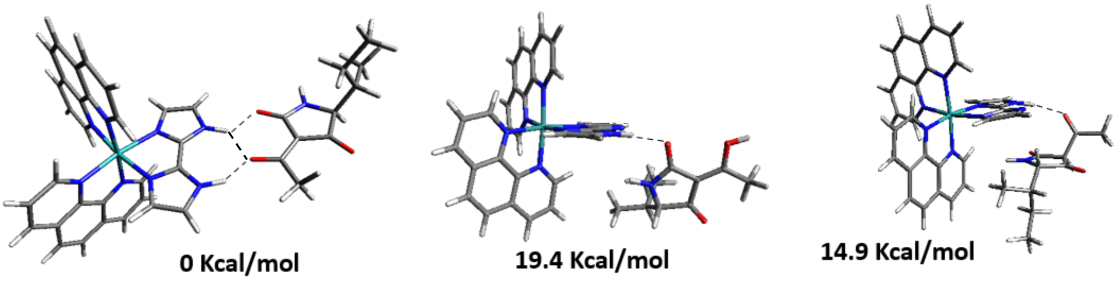

Figure S19. Calculated possible ground state geometries of the $[Ru(phen)_2(bim)]^{2+}$ system, depicting their energies relative to the most stable one (Figure 5b of the main text). The latter accounts for *ca.* 100% of the overall population at 298 K.

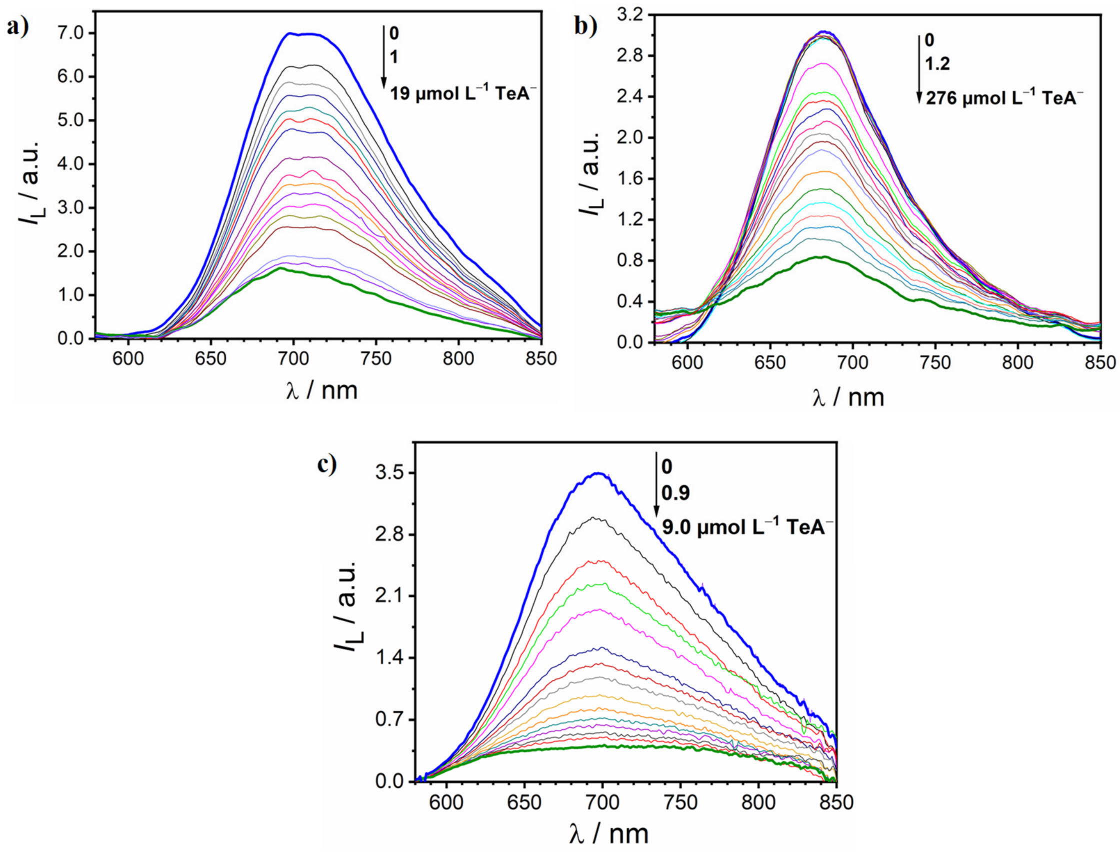

Figure S20. Changes in the luminescence spectra of a) $[Ru(dcb)_2(bim)]^{2+}$ (corrected; λ_{exc} = 520 nm, 10.0 µmol L^{-1}), b) $[Ru(s2b)_2(bim)]^{2-}$ (corrected; λ_{exc} = 510 nm, 12.0 µmol L^{-1}), and c) $[Ru(dab)_2(bim)]^{2+}$ (corrected; λ_{exc} = 500 nm, 9.0 µmol L^{-1}) in DMSO upon the addition of increasing amounts of TeA^- (as TBA salt).

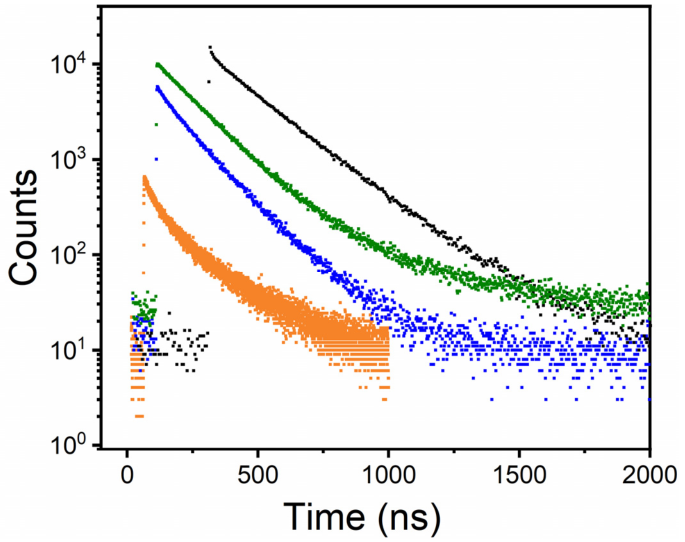

Figure S21. Luminescence decays of the photoexcited $[Ru(phen)_2(bim)]^{2+}$ (black line), $[Ru(dcb)_2(bim)]^{2+}$ (blue line), $[Ru(dab)_2(bim)]^{2+}$ (orange line) and $[Ru(s2b)_2(bim)]^{2-}$ (green line) (λ_{exc} = 463 nm).

Table S1. Energy difference (in kcal mol^{-1}) between the N$^-$···TeAH/AcOH and NH···TeA$^-$/AcO$^-$ forms for different Ru-bim complexes.

Complex	[N$^-$···TeAH]–[NH···TeA$^-$]	[N$^-$···AcOH]–[NH···AcO$^-$]
$[Ru(phen)_2(bim)]^{2+}$	10.35124	3.73802
$[Ru(s2b)_2(bim)]^{2-}$	9.03663	3.60687
$[Ru(dcb)_2(bim)]^{2+}$	24.31312	3.66209

Table S2. Cartesian coordinates (in Å) of the ground state DFT-optimized structures.

**$[Ru(phen)_2(bim)]^{2+}$·····TeA$^-$**				**$[Ru(dcb)_2(bim)]^{2+}$·····TeA$^-$**			
44	1.425882	-0.043836	-0.034134	44	1.500681	-0.054309	-0.024124
6	2.461289	-4.446194	1.810149	6	2.330680	-4.455564	1.839468
6	3.148966	-3.902498	0.701624	6	3.035589	-3.967256	0.738492
6	2.763784	-2.614189	0.266582	6	2.758930	-2.684819	0.264086

7	1.772706	-1.893759	0.873104	7	1.813228	-1.905068	0.858182
6	1.138197	-2.431226	1.916509	6	1.134195	-2.378871	1.919537
6	1.458152	-3.707237	2.412681	6	1.363449	-3.644807	2.440384
6	3.418257	-2.004759	-0.849129	6	3.443122	-2.068942	-0.884858
6	4.455694	-2.687755	-1.523546	6	4.448126	-2.700037	-1.618627
6	5.063065	-2.030831	-2.617261	6	5.036366	-2.029433	-2.691326
6	4.623587	-0.768526	-2.975856	6	4.602833	-0.737667	-3.003429
6	3.582525	-0.161246	-2.252275	6	3.598431	-0.165984	-2.235728
7	2.990279	-0.758428	-1.216414	7	3.026749	-0.810060	-1.200835
1	2.724576	-5.433374	2.177544	6	2.578187	-5.819198	2.394768
1	0.359504	-1.827104	2.367206	1	0.397377	-1.712120	2.348649
1	0.910298	-4.094313	3.264711	1	0.799735	-3.995742	3.296231
1	5.864628	-2.519319	-3.162955	6	6.116965	-2.648274	-3.514790
1	5.067627	-0.234969	-3.808949	1	5.040439	-0.190392	-3.829447
1	3.216504	0.824511	-2.514229	1	3.229144	0.829886	-2.443108
6	4.205096	-4.579398	0.000438	1	3.785183	-4.584208	0.262468
6	4.831500	-3.996897	-1.064825	1	4.774856	-3.698905	-1.364238
6	4.420440	2.816074	2.552031	8	3.525543	-6.491008	1.717348
6	3.667667	3.293534	1.455562	8	6.431494	-3.890902	-3.109184
6	2.842448	2.365746	0.780303	8	6.656728	-2.089648	-4.452616
7	2.749236	1.051653	1.148572	8	1.987146	-6.275828	3.356533
6	3.472200	0.627997	2.186909	1	7.140612	-4.236905	-3.684946
6	4.319272	1.483676	2.912247	1	3.637592	-7.370494	2.127149
6	2.053616	2.784829	-0.336153	6	4.497876	2.687649	2.629363
6	2.094419	4.128341	-0.772492	6	3.742255	3.203689	1.575330
6	1.289522	4.476174	-1.880880	6	2.916122	2.358148	0.838437
6	0.509602	3.503487	-2.481776	7	2.815348	1.032687	1.145340
6	0.529039	2.188457	-1.984570	6	3.530831	0.539353	2.170652
7	1.281235	1.832877	-0.941501	6	4.385681	1.326729	2.932092
1	5.067825	3.494442	3.099250	6	2.099561	2.800397	-0.303691
1	3.371352	-0.418518	2.449150	6	2.062067	4.114685	-0.769258
1	4.882702	1.083397	3.747871	6	1.259113	4.427293	-1.867083
1	1.289676	5.497750	-2.248736	6	0.513693	3.412373	-2.473773
1	-0.120274	3.735862	-3.333394	6	0.594234	2.124726	-1.961527
1	-0.068619	1.404181	-2.434195	7	1.367263	1.820368	-0.902544
6	3.695141	4.654605	0.994525	6	5.368369	3.639375	3.395963
6	2.941172	5.054587	-0.072306	1	3.404608	-0.514465	2.381867
6	-0.611596	1.051999	2.286032	1	4.914165	0.862256	3.757473
6	-1.976339	0.968282	2.468643	6	1.172882	5.813733	-2.414433
7	-0.288336	0.406356	1.117024	1	-0.117930	3.623986	-3.328045
6	-1.438583	-0.058676	0.597321	1	0.037902	1.304150	-2.395890
7	-2.478594	0.266535	1.397588	1	3.815096	4.257987	1.342720
6	-1.340998	-0.781406	-0.642271	1	2.644880	4.889128	-0.289855
7	-0.117414	-0.927695	-1.178176	8	1.936532	6.687661	-1.735944
6	-0.278046	-1.639000	-2.343664	8	0.486023	6.121557	-3.371648
6	-1.619284	-1.918670	-2.507476	8	5.161026	4.836115	3.418890
7	-2.270384	-1.370132	-1.425901	8	6.411015	3.129852	4.079451
1	-3.477446	0.040833	1.256172	1	1.836818	7.570259	-2.142200
1	-3.281341	-1.365785	-1.190847	1	6.546202	2.180643	3.909631
1	0.139316	1.521231	2.903527	6	-0.516427	1.112176	2.281965
1	-2.625543	1.336450	3.246617	6	-1.883229	1.064358	2.458675
1	0.560299	-1.896623	-2.973102	7	-0.205907	0.438639	1.124772
1	-2.150167	-2.448681	-3.283002	6	-1.365500	-0.006147	0.606824
8	-5.068174	0.455246	2.376061	7	-2.398153	0.357817	1.397219
6	-6.228937	0.153753	2.028413	6	-1.281139	-0.748257	-0.622344

6	-6.685847	-0.534678	0.818821	7	-0.060339	-0.924359	-1.157098
8	-4.571512	-0.917442	-0.138866	6	-0.233441	-1.648378	-2.313460
6	-5.829347	-1.030549	-0.200008	6	-1.579608	-1.904401	-2.471903
6	-6.431507	-1.718002	-1.407292	7	-2.219593	-1.329659	-1.397806
7	-7.331923	0.437277	2.791541	1	-3.403489	0.157869	1.254379
6	-8.591370	0.111015	2.138746	1	-3.231570	-1.301925	-1.160549
6	-8.117879	-0.595484	0.852348	1	0.242080	1.574141	2.895527
8	-8.927915	-1.088634	0.046889	1	-2.526328	1.461097	3.227528
6	-9.499316	1.328738	1.852038	1	0.598473	-1.929265	-2.941195
6	-8.790409	2.383333	0.983141	1	-2.120360	-2.434346	-3.240513
6	-9.699662	3.533664	0.536137	8	-4.979445	0.648907	2.341728
1	-7.256453	1.041329	3.598954	6	-6.145689	0.360398	1.999341
1	-10.345609	0.925328	1.278296	6	-6.607300	-0.417790	0.847073
6	-10.044064	1.913853	3.163035	8	-4.502083	-0.805414	-0.127047
1	-7.146288	-1.060353	-1.912649	6	-5.757132	-0.962758	-0.150763
1	-5.635308	-2.004900	-2.099301	6	-6.359860	-1.758935	-1.288722
1	-6.996210	-2.606561	-1.104689	7	-7.248798	0.756594	2.706936
1	-7.931480	2.787627	1.535641	6	-8.503260	0.252148	2.169479
1	-8.380211	1.892299	0.091706	6	-8.035828	-0.521003	0.919145
1	-9.168527	4.201291	-0.152483	8	-8.845500	-1.093309	0.168049
1	-10.042057	4.138728	1.382915	6	-9.544024	1.346535	1.850781
1	-10.587936	3.153395	0.015171	6	-8.997399	2.381754	0.851462
1	-10.811523	2.670670	2.972581	6	-10.042988	3.393741	0.369489
1	-9.248694	2.394639	3.747883	1	-7.154978	1.183479	3.618611
1	-10.495586	1.131205	3.784384	1	-10.381323	0.818234	1.373374
1	-9.159509	-0.612312	2.739761	6	-10.054315	1.991434	3.147030
1	2.969481	6.085752	-0.411135	1	-7.096069	-1.159500	-1.834335
1	4.331182	5.363710	1.515831	1	-5.567114	-2.082983	-1.968213
1	4.497478	-5.569499	0.337167	1	-6.899983	-2.632179	-0.907446
1	5.628781	-4.517264	-1.586914	1	-8.154010	2.912816	1.312210
				1	-8.591022	1.853658	-0.020623
				1	-9.622224	4.046809	-0.404245
				1	-10.398590	4.035059	1.183600
				1	-10.915098	2.884356	-0.060770
				1	-10.895637	2.664709	2.954519
				1	-9.265072	2.580060	3.632951
				1	-10.395016	1.228664	3.857736
				1	-8.955873	-0.469347	2.865210
**$[Ru(s2b)_2(bim)]^{2-}\cdots TeA^-$**				**$[Ru(dab)_2(bim)]^{2+}\cdots TeA^-$**			
44	-1.142993	-0.056007	0.229793	44	1.521657	0.149831	-0.178388
6	-2.594019	-4.197022	-1.838624	6	2.514454	-4.117686	1.953401
6	-3.345499	-3.558778	-0.859823	6	3.197004	-3.660516	0.828297
6	-2.875479	-2.359716	-0.316954	6	2.872609	-2.418905	0.275767
7	-1.700664	-1.813056	-0.731976	7	1.896989	-1.640243	0.815260
6	-0.978879	-2.439653	-1.680813	6	1.236093	-2.081259	1.901927
6	-1.389082	-3.631870	-2.261240	6	1.514296	-3.302662	2.497753
6	-3.589064	-1.594520	0.718759	6	3.541279	-1.850336	-0.907964
6	-4.810381	-1.990357	1.270706	6	4.577607	-2.490820	-1.591801
6	-5.402396	-1.195459	2.243847	6	5.156556	-1.883474	-2.704074
6	-4.772409	-0.016219	2.646793	6	4.668341	-0.631576	-3.099610
6	-3.560554	0.318910	2.059498	6	3.637209	-0.046230	-2.380213
7	-2.973579	-0.448523	1.120883	7	3.077790	-0.634430	-1.306019
1	-0.055086	-1.954510	-1.970703	1	0.471854	-1.420552	2.292026
1	-0.780008	-4.101250	-3.025218	1	0.953390	-3.607565	3.375415
1	-5.208422	0.632293	3.397931	1	5.082033	-0.110882	-3.957392

1	-3.030004	1.220576	2.337988	1	3.234264	0.919685	-2.658178
1	-4.280327	-3.997868	-0.536581	1	3.974500	-4.271977	0.392048
1	-5.304651	-2.899613	0.954541	1	4.937724	-3.456090	-1.264137
16	-3.160308	-5.761624	-2.555478	6	4.482918	3.137229	2.272039
8	-4.515455	-5.990257	-1.977184	6	3.730075	3.553552	1.176899
8	-2.142082	-6.763461	-2.120085	6	2.907297	2.641926	0.510466
8	-3.163110	-5.521991	-4.028841	7	2.821991	1.344167	0.910525
16	-6.971329	-1.690789	3.002178	6	3.552220	0.940741	1.967458
8	-6.627081	-1.959757	4.430364	6	4.386681	1.797710	2.669119
8	-7.404350	-2.902765	2.249512	6	2.077088	2.994498	-0.654791
8	-7.868621	-0.511968	2.820872	6	2.022657	4.275758	-1.209392
6	-3.009485	3.220528	-2.815522	6	1.210795	4.519085	-2.315654
6	-2.376160	3.587224	-1.633664	6	0.469590	3.451421	-2.837794
6	-1.871488	2.591409	-0.794641	6	0.564640	2.202339	-2.242166
7	-1.989008	1.272959	-1.117265	7	1.349968	1.969332	-1.173969
6	-2.609034	0.928667	-2.261554	1	3.451085	-0.100419	2.246986
6	-3.132657	1.868852	-3.139290	1	4.951354	1.417799	3.514489
6	-1.185393	2.866932	0.478580	1	-0.178851	3.583561	-3.698029
6	-0.964916	4.152572	0.980486	1	0.007996	1.351686	-2.615609
6	-0.308132	4.299417	2.195735	1	3.788622	4.582174	0.850064
6	0.117274	3.165862	2.891636	1	2.604343	5.083534	-0.787358
6	-0.132816	1.918190	2.337445	6	-0.523120	1.396197	2.062572
7	-0.769552	1.765537	1.160395	6	-1.885402	1.306565	2.260061
1	-2.678623	-0.132375	-2.465663	7	-0.196011	0.665672	0.945615
1	-3.631189	1.548864	-4.046700	6	-1.341827	0.144839	0.470921
1	0.629607	3.243783	3.843707	7	-2.382440	0.516088	1.250199
1	0.171302	1.004773	2.833253	6	-1.241039	-0.671158	-0.709182
1	-2.290878	4.635021	-1.376586	7	-0.019714	-0.837932	-1.244547
1	-1.295675	5.033778	0.446357	6	-0.178049	-1.641693	-2.348748
16	-3.626120	4.492626	-3.948879	6	-1.515401	-1.956680	-2.476092
8	-3.794583	5.712700	-3.108305	7	-2.166607	-1.335753	-1.434589
8	-4.904949	3.941911	-4.483964	1	-3.378125	0.262589	1.135163
8	-2.554741	4.621226	-4.982345	1	-3.174598	-1.335958	-1.186193
16	0.011230	5.949932	2.871257	1	0.223635	1.923760	2.636604
8	1.499631	6.071646	2.875332	1	-2.536077	1.724044	3.011475
8	-0.667377	6.893153	1.937082	1	0.658857	-1.933385	-2.965109
8	-0.584775	5.919923	4.239568	1	-2.043423	-2.555401	-3.201975
6	1.436889	0.615491	-1.679446	8	-4.965263	0.730288	2.239461
6	2.771000	0.268944	-1.629803	6	-6.109807	0.300728	1.983392
7	0.798873	0.042545	-0.605772	6	-6.542339	-0.607650	0.918474
6	1.731244	-0.637362	0.085687	8	-4.462243	-0.839203	-0.155741
7	2.936508	-0.518699	-0.514419	6	-5.691006	-1.130933	-0.090918
6	1.284552	-1.329073	1.264977	6	-6.262788	-2.067912	-1.134069
7	-0.016747	-1.229596	1.585708	7	-7.213608	0.638781	2.720924
6	-0.199363	-1.963072	2.734271	6	-8.429778	-0.040235	2.299861
6	1.013268	-2.505881	3.106422	6	-7.944301	-0.858241	1.085385
7	1.933127	-2.094238	2.168943	8	-8.723077	-1.574602	0.431179
1	3.834954	-0.931980	-0.212668	6	-9.601185	0.906384	1.961741
1	2.950118	-2.293093	2.102876	6	-9.224737	1.908535	0.855836
1	0.908166	1.222827	-2.398425	6	-10.400703	2.755951	0.357109
1	3.603644	0.505694	-2.272319	1	-7.117102	1.128631	3.600136
1	-1.166042	-2.050274	3.206788	1	-10.397798	0.254475	1.576317
1	1.290168	-3.134545	3.938405	6	-10.113400	1.597134	3.233397
8	5.647963	-0.815082	-1.016953	1	-7.086405	-1.590318	-1.675247
6	6.646852	-1.361145	-0.503069	1	-5.475866	-2.357127	-1.835668

6	6.729333	-2.212650	0.686925
8	4.461522	-2.105017	1.303289
6	5.628062	-2.541301	1.521676
6	5.846012	-3.441030	2.720066
7	7.909155	-1.239375	-1.018423
6	8.917142	-1.996090	-0.292976
6	8.090453	-2.635088	0.842286
8	8.619348	-3.368656	1.696968
6	10.099733	-1.148825	0.224245
6	9.627727	-0.010946	1.147045
6	10.769754	0.759128	1.819747
1	8.068619	-0.783602	-1.906478
1	10.711971	-1.841025	0.819455
6	10.948289	-0.641327	-0.950202
1	6.581938	-3.003777	3.403291
1	4.897680	-3.589544	3.243271
1	6.254794	-4.408583	2.410514
1	9.004332	0.683714	0.568670
1	8.980115	-0.431459	1.926874
1	10.374930	1.493222	2.532051
1	11.381913	1.303243	1.091951
1	11.431434	0.079273	2.372221
1	11.872008	-0.169063	-0.600968
1	10.400576	0.103967	-1.541855
1	11.228255	-1.465360	-1.617755
1	9.315582	-2.805907	-0.921283

1	-6.685376	-2.961442	-0.662507
1	-8.426005	2.565176	1.225163
1	-8.803348	1.357388	0.005413
1	-10.094306	3.385207	-0.486911
1	-10.788683	3.419259	1.138159
1	-11.228106	2.119714	0.016837
1	-11.030930	2.161913	3.040101
1	-9.368885	2.301326	3.627571
1	-10.334199	0.862791	4.017653
1	-8.762490	-0.746507	3.074415
6	5.385081	4.071085	3.036861
1	6.412885	3.693076	3.018015
1	5.055879	4.128834	4.079357
6	1.101602	5.875314	-2.958640
1	0.071111	6.242282	-2.911094
1	1.391099	5.827384	-4.013470
6	6.273405	-2.520875	-3.485823
1	5.962841	-2.708439	-4.518963
1	7.149070	-1.864377	-3.514030
6	2.802545	-5.454568	2.585846
1	1.943662	-6.119082	2.444478
1	2.972382	-5.329717	3.660017
8	1.965362	6.790746	-2.268023
6	1.984039	8.065417	-2.717060
8	1.310723	8.441095	-3.660244
6	2.925082	8.915247	-1.908089
1	3.938883	8.505035	-1.962728
1	2.918810	9.937214	-2.288476
1	2.622971	8.908794	-0.855642
8	5.330249	5.369141	2.426959
6	5.996810	6.428417	2.950635
8	5.908723	7.491017	2.363993
6	6.790274	6.222424	4.214983
1	7.287820	7.157054	4.473665
1	6.134193	5.923735	5.040071
1	7.540172	5.434995	4.086629
8	3.968511	-6.018109	1.966342
6	4.419419	-7.254447	2.296166
8	5.419982	-7.654724	1.730641
6	3.657590	-8.044531	3.328901
1	4.170315	-8.992047	3.494264
1	3.592443	-7.497224	4.275150
1	2.634793	-8.243067	2.989953
8	6.629295	-3.764147	-2.862428
6	7.616698	-4.477302	-3.448375
8	8.190140	-4.103038	-4.455895
6	7.888515	-5.752010	-2.697802
1	8.692802	-6.303283	-3.186016
1	8.168040	-5.524711	-1.663826
1	6.982913	-6.366805	-2.667013

Table S3. Global analysis of the luminescence lifetimes of $[Ru(phen)_2(bim)]^{2+}$ (12.0 µmol L^{-1}) at (25 ± 1) ºC in air-equilibrated DMSO upon the addition of increasing amounts of TeA^{-}.[a]

[TeA] (µmol L^{-1})	τ_1/ns (B_1) (%)	τ_2/ns (B_2) (%)	τ_m/ns[c]
2.4	68 (1447) (6)	164 (8557) (94)	150
6.0	68 (1682) (8)	164 (8156) (92)	148
9.6	68 (2644) (14)	164 (6643) (86)	137
12.0	68 (3552) (30)	164 (3242) (70)	114
24.0	68 (2807) (40)	164 (2329) (60)	112

[a] To determine the luminescence lifetimes, the decays are fitted to the function $I_L = B_0 + \sum_i B_i \tau_i$, being i the number of exponentials required to achieve a global $\chi^2 < 1.08$. [b] The relative contributions to the overall luminescence have been calculated as $\%_i = B_i \tau_i / \sum_i B_i \tau_i$. [c] Pre-exponentially weighted average luminescence lifetime: $\tau_m = \sum_i B_i \tau_i / \sum_i B_i$; estimated uncertainties of the lifetimes: τ_i ± 3%; τ_m ± 1.5%.

REFERENCE

(1) Orellana, G.; Álvarez-Ibarra, C.; Santoro, C. Hydrogen-1 and carbon-13 NMR coordination-induced shifts in a series of tris(α-diimine)ruthenium(II) complexes containing pyridine, pyrazine, and thiazole moieties. *Inorg. Chem.* **1988**, *27*, 1025–1030.
